# Er:YAG and Nd:YAG-based low-level laser therapy (LLLT) with medical collagen improve third-molar extraction wound healing: a randomized controlled trial

**DOI:** 10.1007/s10103-025-04763-7

**Published:** 2025-12-08

**Authors:** Zhantang Yuan, Zhihui Xu, Xia Wan, Tonghan Zhang

**Affiliations:** 1 Department of Oral Emergency, Hospital of Stomatology, Zhongshan City, No. 73, Hubin Road, Shiqi District, Guangdong 528400 Zhongshan, China; 2 Department of Anesthesiology, Zhongshan People’s Hospital, No. 2, Sun Wen East Road, Shi Qi District, Guangdong 528400 Zhongshan, China; 3 Faculty of Chinese Medicine, Macau University of Science and Technology, Avenida Wai Long, Taipa, Macao SAR 999078 Macau, China; 4 Oral and Maxillofacial Surgery, Hospital of Stomatology, Zhongshan City, No. 73, Hubin Road, Shi Qi District, Guangdong 528400 Zhongshan, China

**Keywords:** Impacted teeth, Er:YAG laser, Nd:YAG laser, Low-level laser therapy, Collagen, Postoperative complications

## Abstract

To evaluate whether Er: YAG surgery and Nd: YAG-based low-level laser therapy (LLLT), combined with medical collagen, improve early postoperative outcomes after extraction of impacted mandibular third molars. Adults undergoing surgical removal of mesioangular or horizontal impacted mandibular third molars were randomized equally (1:1:1:1) to blank control (BCG), collagen only (COL), lasers only (LAS), or collagen plus lasers (COL + LAS). **Primary endpoints** were pain (VAS; spontaneous and swallowing; POD1/3/7), swelling (POD1/3/7), trismus (POD1/3/7), and early mucosal healing (POD3/7). **Secondary endpoints** were bleeding (30 min post-extraction only), cutaneous induration/ecchymosis (POD7), alveolar osteitis (POD3/7), and local cytokines (IL-1β, IL-6, TNF-α; POD3/7). In 120 patients, **COL + LAS** was superior: less pain, swelling, and trismus at **POD1/3** and higher early mucosal-healing rates at **POD3/7** than **BCG**, **COL**, or **LAS**; by **POD7**, between-group differences in pain, swelling, and trismus were minimal. All active arms reduced **30-min bleeding** versus BCG. At **POD7**, cutaneous induration was lowest with COL + LAS (ecchymosis not significant). Cytokines declined overall; **IL-1β** and **TNF-α** were lowest with COL + LAS at **POD3/7**, whereas **IL-6** decreased similarly across active arms. Er: YAG surgery plus Nd: YAG-based LLLT with medical collagen improved short-term recovery after impacted mandibular third-molar surgery.

**Supplementary Information:**

The online version contains supplementary material available at 10.1007/s10103-025-04763-7.

## Introduction

Impacted teeth are teeth that fail to erupt completely or at all because of resistance from soft tissues, bone, or adjacent teeth; the condition most commonly involves maxillary and mandibular third molars [1]. Third molars are linked to dental caries, pericoronitis, damage to adjacent teeth and periodontal tissues, and an increased risk of cysts and tumors [2].Whether asymptomatic third molars should be removed prophylactically remains debated; current recommendations favor individualized risk–benefit evaluation [3]. In clinical practice, mandibular third-molar extraction is among the most frequent dentoalveolar procedures [4]. Nevertheless, postoperative pain, bleeding, swelling, trismus, and impaired wound healing remain common [5]. As living standards improve, patient expectations for minimally invasive, painless extraction continue to increase. Therefore, safe and effective strategies are needed to minimize adverse events after extraction of impacted teeth [6].

In recent years, several adjunctive materials for filling extraction sockets have demonstrated efficacy in reducing complications, including hyaluronic acid [7], medical collagen [7, 8], platelet-rich fibrin (PRF) [9], and guided tissue regeneration using resorbable or non-resorbable membranes with bone substitutes [10]. Recent clinical reports indicate that oral probiotics can modulate the oral microbiome and inflammatory milieu, thereby improving wound healing and postoperative comfort after oral surgery [11, 12].Medical collagen—typically produced by low-temperature lyophilization of bovine Achilles-tendon type I collagen, which is homologous to human type I collagen—is widely used for tissue repair. As a scaffold, collagen forms a stable, cross-linked network that supports cell migration and proliferation, thereby promoting tissue growth and wound healing. Owing to its ability to dampen postoperative inflammation, shorten the duration of post-extraction pain, lessen trismus, and accelerate alveolar-bone remineralization [13], medical collagen has become a commonly used adjunct for socket management in oral and maxillofacial surgery [7].

The Er: YAG laser (2,940 nm) is strongly absorbed by water and hydroxyapatite, and ablates tissue via a water-mediated, thermomechanical process in which rapid vaporization produces micro-explosions [14]. Consequently, compared with Nd: YAG and diode lasers, Er: YAG irradiation minimizes collateral thermal effects at the tissue surface. This mechanism—often described as “cold ablation”—reduces thermal injury to alveolar bone relative to conventional turbines [14]. The Er: YAG laser also exerts photobiomodulatory effects, promoting type I collagen formation and osteogenesis [15]. In addition, Er: YAG irradiation provides biostimulatory and antimicrobial actions on wound surfaces [16].

The Nd: YAG laser (1,064 nm) penetrates deeply into tissue and is therefore of particular interest for promoting healing. Its primary mechanism is melanin-mediated absorption of laser energy, which generates heat and produces a photothermal effect [17]. Pigmented bacteria, such as Porphyromonas gingivalis, are susceptible to this thermal action, yielding bactericidal effects [18]. The Nd: YAG laser also inactivates bacterial virulence factors, including endotoxin (lipopolysaccharide), thereby reducing the risk of postoperative infection [19]. Owing to its penetration depth, Nd: YAG irradiation can induce cellular coagulation—including of neural elements—which decreases nociceptor sensitivity and contributes to hemostasis and analgesia [20].

This study evaluates the therapeutic efficacy and clinical feasibility of combining Er: YAG and Nd: YAG lasers with medical collagen for post-extraction management of sockets after impacted third-molar removal. Our objective is to optimize minimally invasive extraction workflows, reduce postoperative complications, and improve patient comfort.

## Materials and methods

### Ethics and registration

The study was approved by the Ethics Committee of the Hospital of Stomatology, Zhongshan City (Approval No. [2024] Research Project No. 7; March 18, 2024) and conducted in accordance with the Declaration of Helsinki. The trial was registered with the Chinese Clinical Trial Registry (PID 248812). Written informed consent was obtained from all participants.

### Trial design and setting

Prospective, single-center, four-arm, parallel-group, superiority randomized controlled trial with equal allocation (1:1:1:1), conducted from October 2023 to June 2024 and reported in accordance with CONSORT. Only one index mandibular third molar per participant was included (parallel design; no split-mouth).

### Participants (Eligibility Criteria)

#### Inclusion

Adults scheduled for removal of a mandibular third molar with mesioangular or horizontal impaction (Pell–Gregory class B/C) requiring mucoperiosteal flap elevation; no acute infection; medically fit; able to comply with follow-up.

#### Exclusion

Uncontrolled systemic disease affecting peri-operative risk (e.g., hypertension, heart disease, diabetes, hyperthyroidism, hematologic disorders); pregnancy or menstruation; acute pericoronitis/infection within 7 days; use within 7 days of anticoagulants/antiplatelets, hemostatics, psychotropics, analgesics, or oral contraceptives; collagen allergy; jaw cysts/tumors; periodontitis with bleeding on probing or probing depth > 3 mm and/or attachment loss; other conditions affecting hemostasis or wound healing.

### Interventions

**Randomization** was generated pre-incision using a computer-based random-number table and implemented by personnel independent of the surgeons via sequentially numbered, opaque, sealed envelopes. Participants were assigned in equal numbers (*n* = 30/arm) to BCG, COL, LAS, or COL + LAS.

**Fig. 1:**
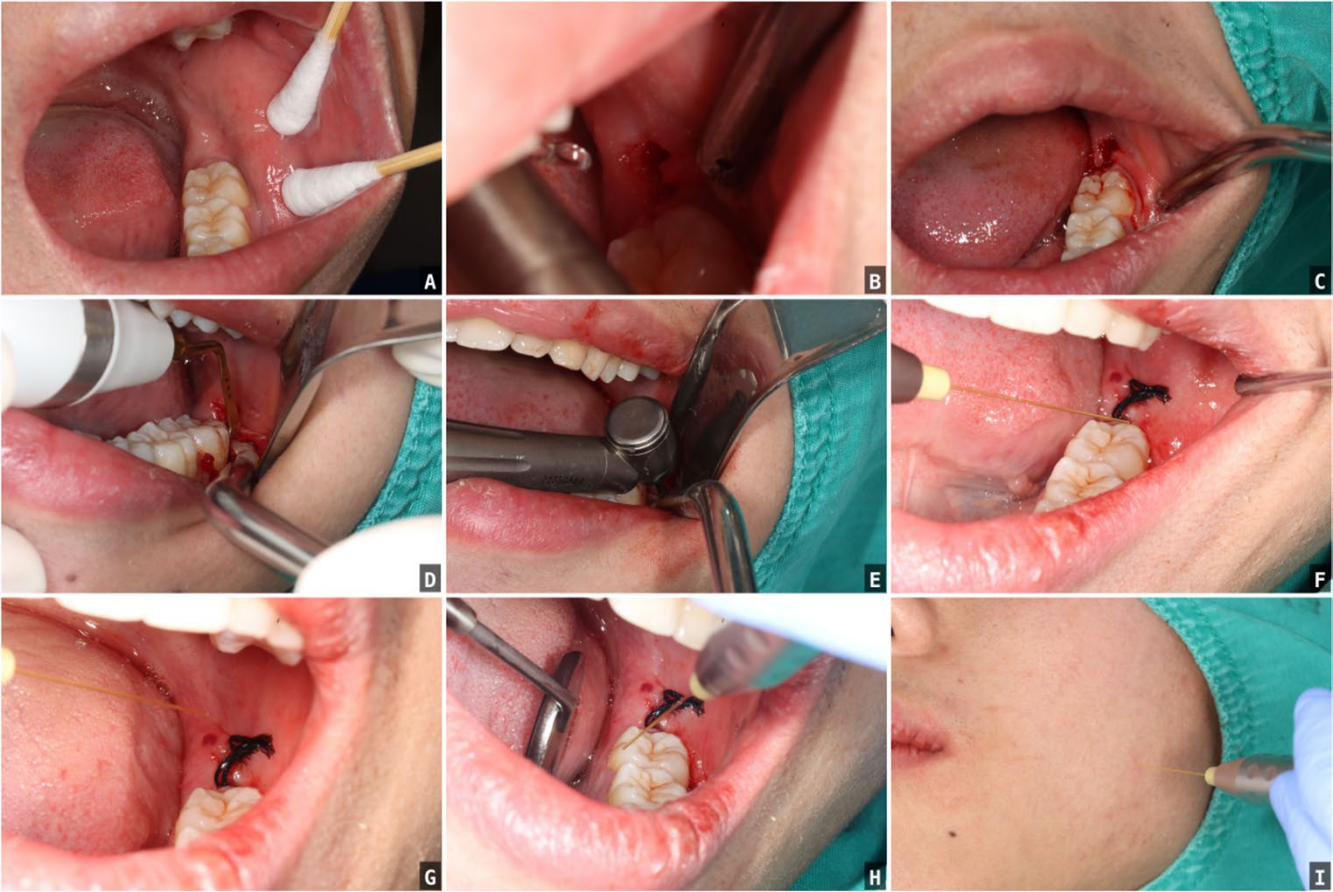
Surgical workflow for impacted mandibular third-molar extraction with adjunctive lasers and collagen. **A**. Pre-operative photograph of the operative site. **B**-**C**.Er: YAG laser gingival incision. **D**. Piezoelectric osteotomy (PIEZOTOME) for conservative bone removal. **E**. Tooth sectioning with a high-speed, motor-driven electric contra-angle handpiece. F. Nd: YAG low-level laser photobiomodulation applied to the operative site. **G**. Photobiomodulation to anterior tonsillar pillar. **H**. Photobiomodulation to the pterygomandibular region. **I**. Photobiomodulation to the masseter region overlying the operative side

**Fig. 2:**
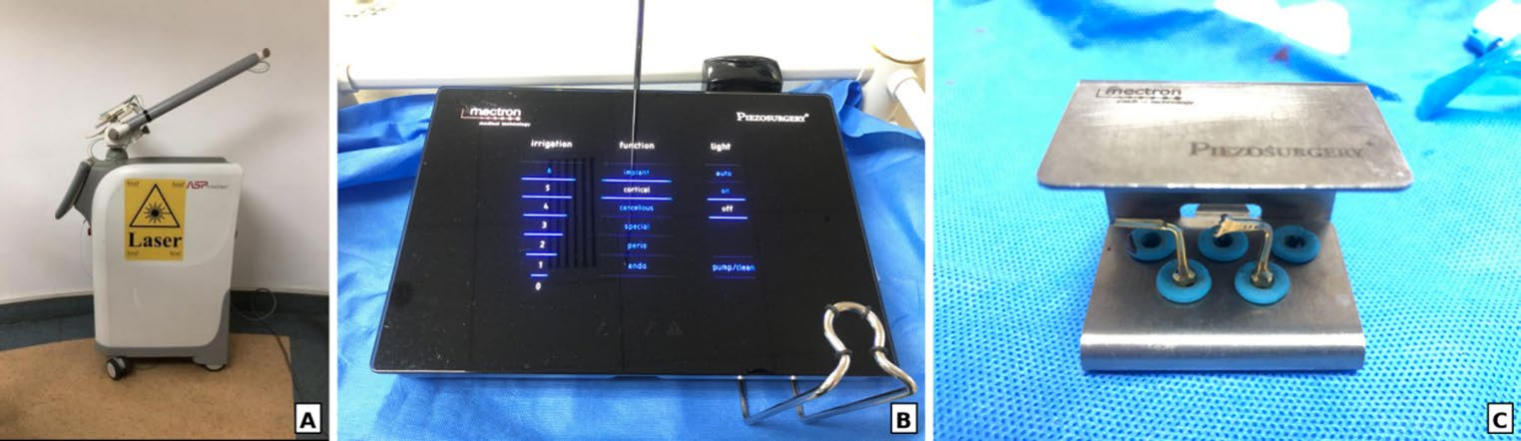
Dual-wavelength laser and ultrasonic osteotomy systems. **A**, Er: YAG & Nd: YAG laser unit (Fotona Light Walker). **B**, Piezoelectric bone surgery console (Mectron Piezosurgery). **C**, Piezoelectric bone surgery handpieces and tips set

Surgical Procedure(Figs. 1 and 2)

1. Asepsis: 0.2% chlorhexidine rinse, perioral 7.5% povidone–iodine, sterile draping.
2. Anesthesia: inferior alveolar, lingual, and buccal nerve blocks with articaine/epinephrine.
3. Incision/flap: oblique mesiobuccal incision at the second molar with a distal relieving incision at the third; full-thickness mucoperiosteal flap.
4. Ostectomy: PIEZOTOME (US1) to expose the maximal tooth circumference.
5. Space creation: UC1/UC5 tips.
6. Sectioning/delivery: 45° high-speed contra-angle handpiece and elevators. All procedures were performed by a single surgeon.

**BCG** (blank control): socket curettage and suturing; sham laser (device off with identical handling and sound cues).

**COL** (collagen only): as BCG plus placement of a collagen cylinder (7 × 15 mm) in the socket, then suturing.

**LAS** (lasers only):

- Er: YAG—incision: 1.0-mm fiber, SP, 120 mJ, 15 Hz (2.4 W); water 4, air 4. Gingival separation: 0.5-mm fiber, MSP, 40 mJ, 20 Hz; water 6, air 4.
- Er: YAG—socket degranulation: MSP, 80 mJ, 20 Hz (1.6 W); water 8, air 4; sweep all socket walls for 5 min.
- Nd: YAG LLLT: MSP, 15 Hz, 1.5 W; handpiece ~ 1 cm from the gingiva; sites: distal-incision center, mesial, distal, buccal, lingual; mesial incision; anterior tonsillar pillar; pterygomandibular region; masseter; ~5 min per site; flap sutured.

**COL + LAS** (collagen plus lasers): identical Er: YAG steps and Nd: YAG LLLT as above, then collagen cylinder placement and suturing.

### Outcomes and assessments

#### Primary endpoints

**Pain** (VAS 0–10; POD1/3/7): two domains recorded separately on a 10-cm VAS (0 = no pain; 10 = worst imaginable): spontaneous pain at rest and swallowing-evoked pain during deglutition (standardized instructions).

Swelling (POD1/3/7): Marković–Todorović [21] linear distance (from the midline-inferior chin point to the junction of the earlobe’s lower border with cheek skin), measured three times and averaged; grading by change from baseline: Grade I < 2 mm, Grade II 2–4 mm, Grade III >4 mm.

**Trismus** (POD1/3/7): maximum interincisal distance (mm) measured with a caliper (×3, averaged) and reported as change from baseline (ΔMID).

Early mucosal healing [22, 23] (POD3 and POD7; 3-grade rubric):

- POD3 — Good: pink/normal margin, socket contraction, no edema, free opening; Fair: mild marginal erythema limited to the wound edge with slight discomfort, modest granulation; Poor: deep erythema extending to attached gingiva/adjacent tooth, edema or clot breakdown/absent granulation, mouth-opening discomfort.
- POD7 — Good: complete epithelial coverage, no erythema/edema, granulation fills the socket; Fair: slight residual erythema with near-complete granulation fill; Poor: deep erythema with marginal/attached-gingival swelling, scant granulation, partially empty socket.

#### Secondary endpoints

**Bleeding** (30 min post-op only; 3-level): 0 = none (gauze minimally stained, no active socket bleeding); 1 = mild (gauze largely stained, blood-streaked saliva, no active socket bleeding); 2 = marked (gauze fully soaked with active oozing and/or visible clots).

**Cutaneous induration/ecchymosis** (POD7): presence/absence of cheek ecchymosis or palpable induration at mandibular border/buccal tissues.

Alveolar osteitis (POD3 and POD7): present/absent per standard criteria—severe pain peaking 48–72 h post-extraction with fetor and an empty or disintegrating clot.

**Local cytokines** (POD3 and POD7): socket exudate collected after isolation and air-drying: four paper points placed for 30 s (mesiobuccal and mesiolingual aspects), pooled into EP tubes and stored at − 80 °C; eluates (PBS, 10,000 rpm, 15 min) assayed by ELISA for IL-1β, IL-6, and TNF-α per manufacturer instructions.

### Sample size determination

Pilot day-3 rates (Oct 2023–Feb 2024) for spontaneous pain, swelling, and trismus informed power. With two-sided α = 0.05 and 90% power (PASS 2023), required totals for the four-arm design were 76, 72, and 84, respectively. Allowing 20% attrition, the largest requirement yielded *N* = 104 (26/group); the target enrollment was *N* = 120.

### Blinding

The BCG group received a sham laser (device off with identical handling/sound cues). For safety, surgeons were aware of energy delivery; participants, outcome assessors, and data analysts were blinded.

### Statistical analysis

Normality (Shapiro–Wilk) and homogeneity of variance (Levene) were assessed. Continuous outcomes were analyzed with linear mixed-effects models (fixed: group, time, group×time; random: subject intercept). Ordinal outcomes used proportional-odds models; binary outcomes used χ² or Fisher’s exact tests. Multiplicity was controlled with Tukey (parametric) or Holm–Bonferroni/Dunn procedures, as appropriate. Two-sided *P* < 0.05 was considered significant. Analyses were performed in SPSS v27.0.

## Results

### Participant flow

**Fig. 3:**
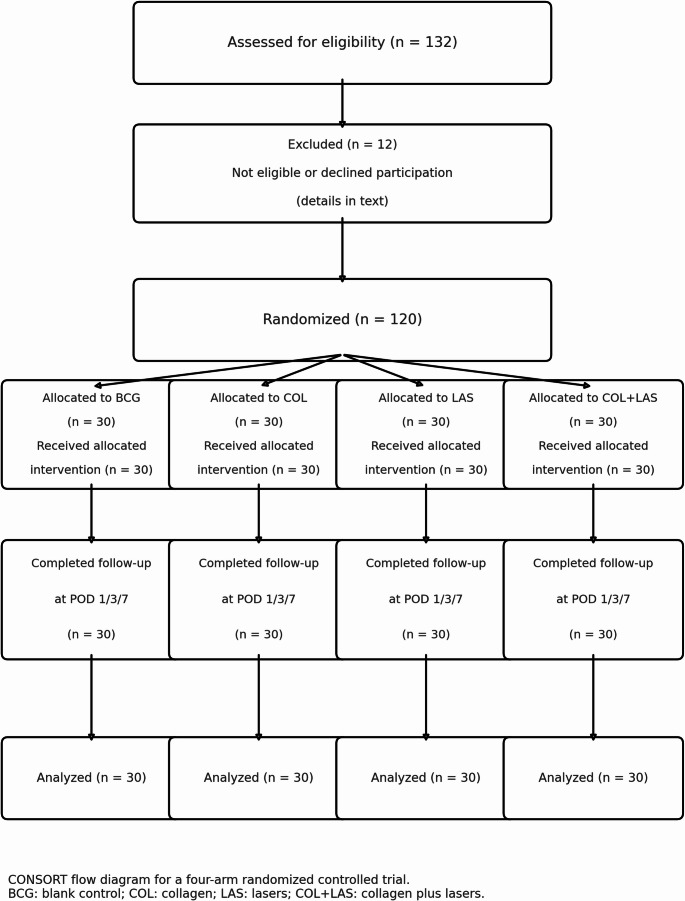
CONSORT participant flow diagram

Of 132 patients screened, 120 were randomized and included in the analysis (BCG *n* = 30; COL *n* = 30; LAS *n* = 30; COL + LAS *n* = 30) **(**Fig. 3**)**.

### Baseline characteristics

Groups were comparable for age, sex, side, and impaction depth (all *P* > 0.05) **(**Table 1**)**.

**Table 1:** Baseline characteristics of participants by treatment group

Group	BCG	COL	LAS	COL+LAS	Total	*P*value
Age (years)	28.00±6.48	29.23±7.48	31.03±7.27	29.60±6.57	29.47±6.96	0.977
Sex						
Male	14	15	16	13	58	0.881
Female	16	15	14	17	62	
Position						
Left	14	17	15	13	59	0.761
Right	16	13	15	17	61	
Type of Obstruction						
Median obstruction	15	15	15	15	60	1
Low obstruction	15	15	15	15	60	
	30	30	30	30	120	

### Primary endpoints

#### Pain (VAS)

##### Spontaneous pain

**Fig. 4:**
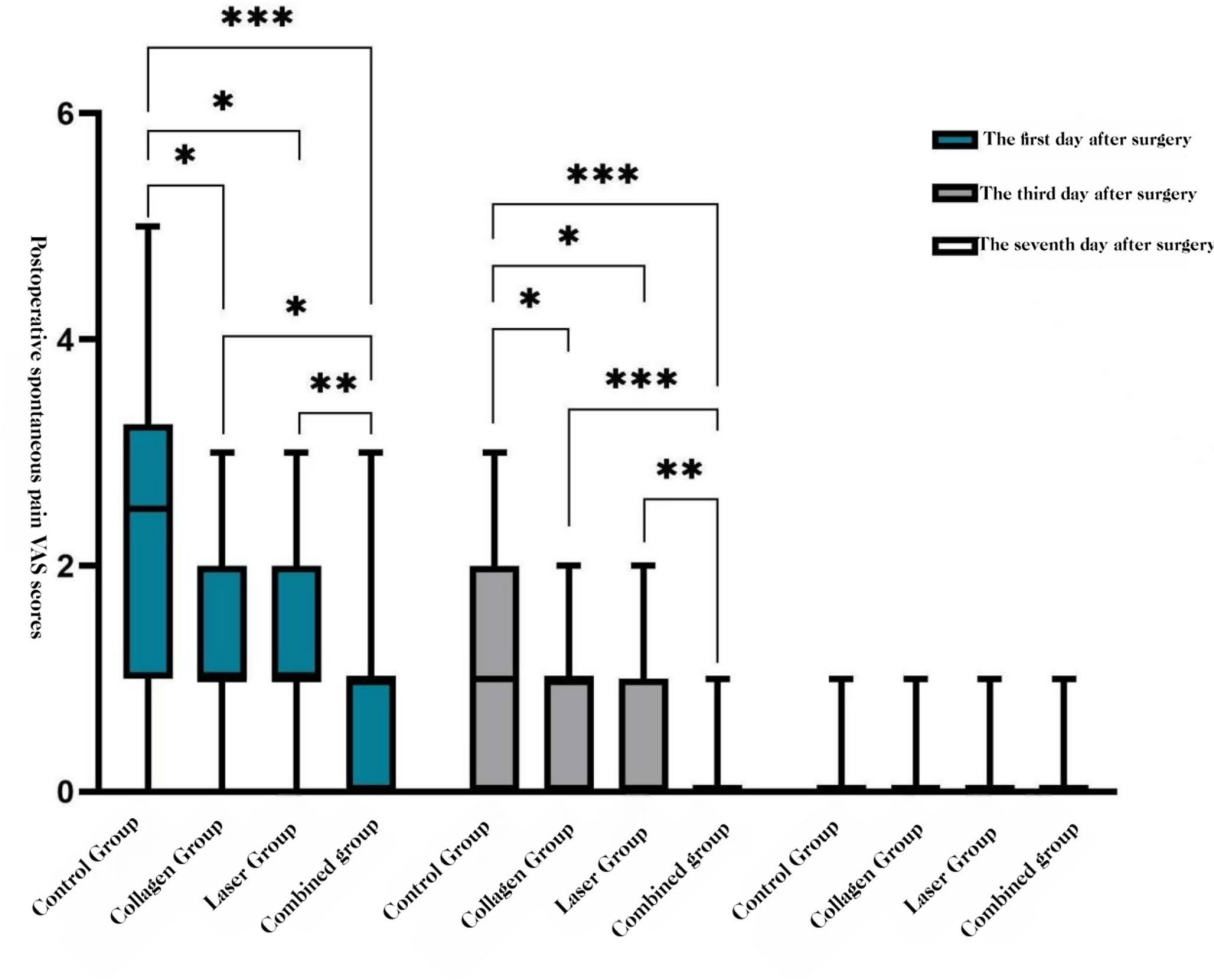
Spontaneous pain VAS by group (POD 1, 3, 7): box-and-whisker plots.

**Fig. 5:**
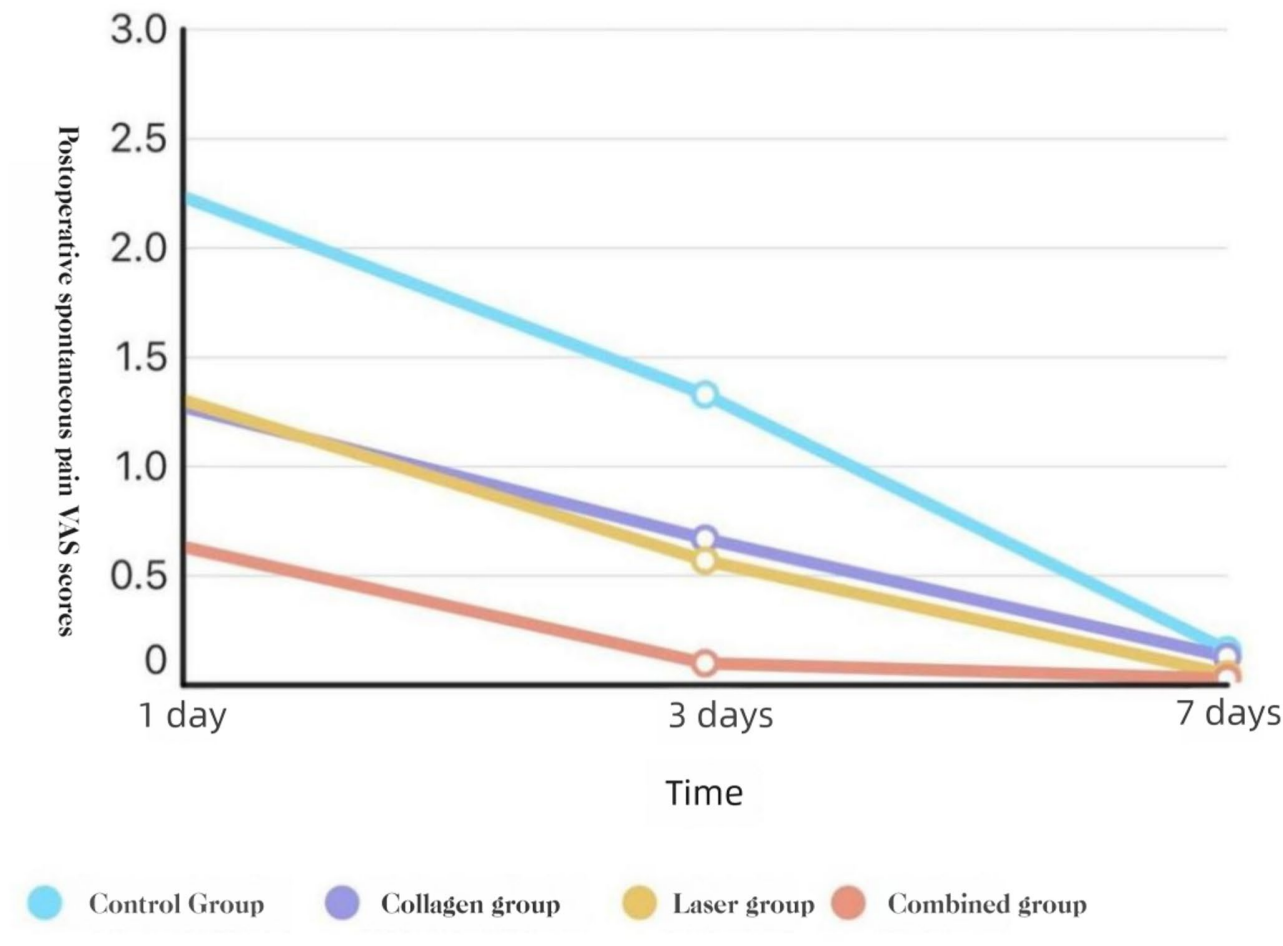
Spontaneous pain VAS over time by group (POD 1, 3, 7):line graph of group means

At POD 1 and POD 3, COL + LAS was lower than all other groups, and COL and LAS were each lower than BCG (all *P* < 0.05). At POD 7, no group differences were observed (*P* > 0.05).**See** Figs. 4 and 5.

##### Swallowing-evoked pain

At POD 3, COL + LAS and LAS were lower than COL and BCG, and COL was lower than BCG (all *P* < 0.05). At POD 1 and POD 7, no group differences were observed (*P* > 0.05).**See** Figs. ,6, and 7

**Fig. 6:**
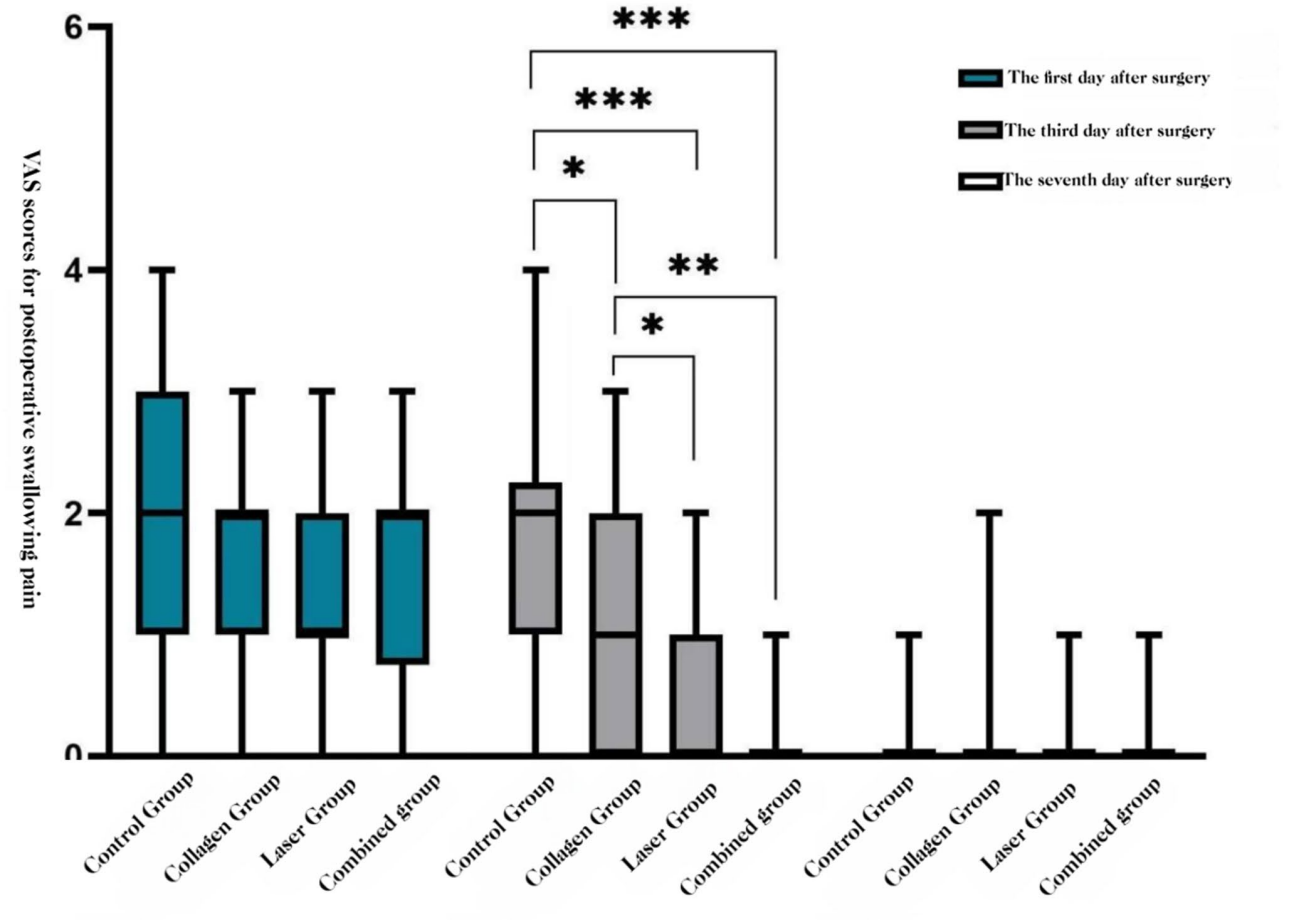
Swallowing-evoke VAS by group (POD 1, 3, 7): box-and-whisker plots

**Fig. 7:**
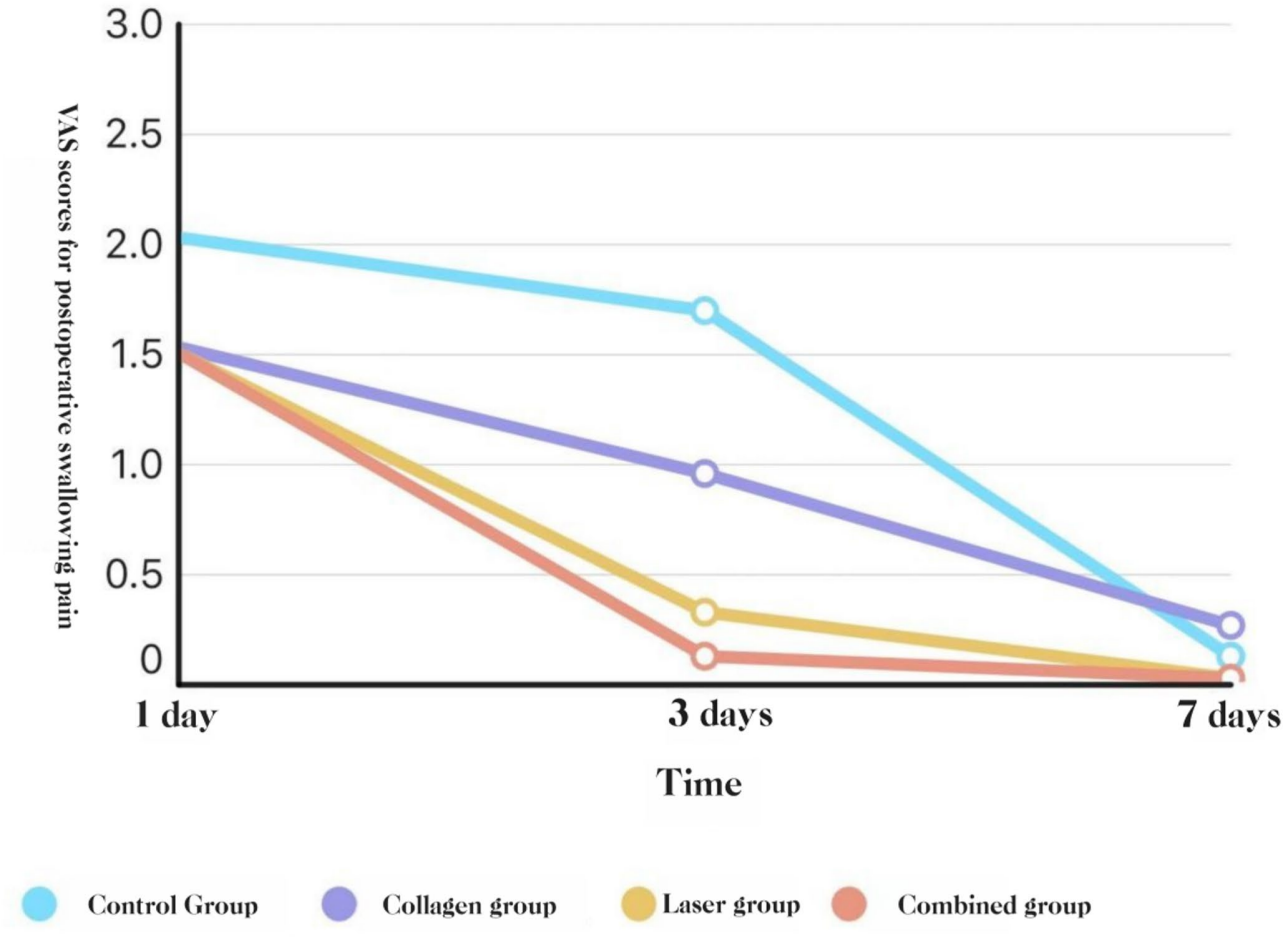
Swallowing-evoke VAS over time by group (POD 1, 3, 7): line graph of group means. * P < 0.05; ** P < 0.01; *** P < 0.001

### Swelling (graded)

POD 1: COL + LAS was lower than COL, LAS, and BCG (*P* < 0.05).

POD 3: COL + LAS was lower than all other groups, and COL and LAS were each lower than BCG (all *P* < 0.05).

**Fig. 8:**
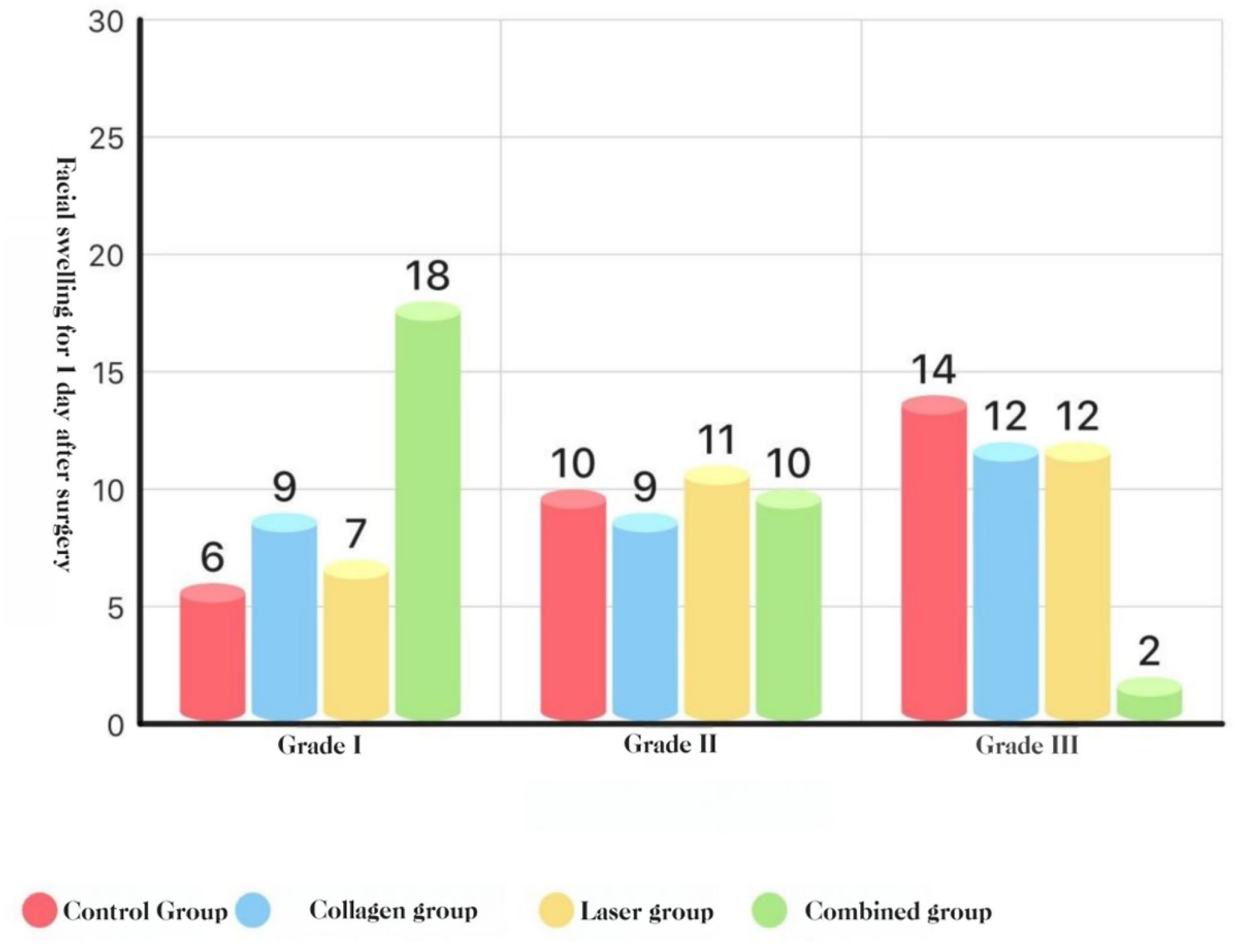
Distribution of facial swelling at POD 1 (Marković–Todorović grades): histogram

**Fig. 9:**
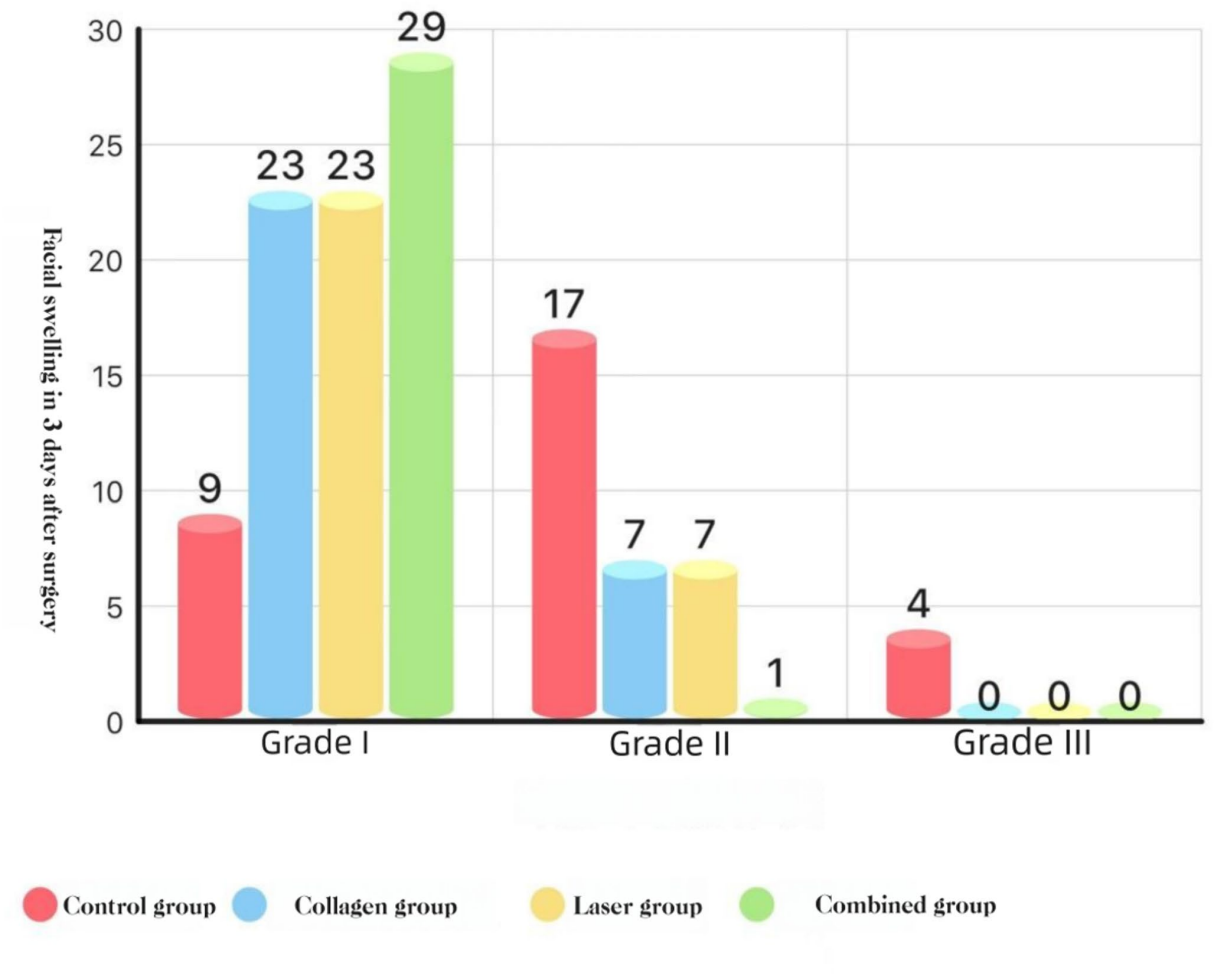
Distribution of facial swelling at POD 3(Marković–Todorović grades): histogram

**Fig. 10:**
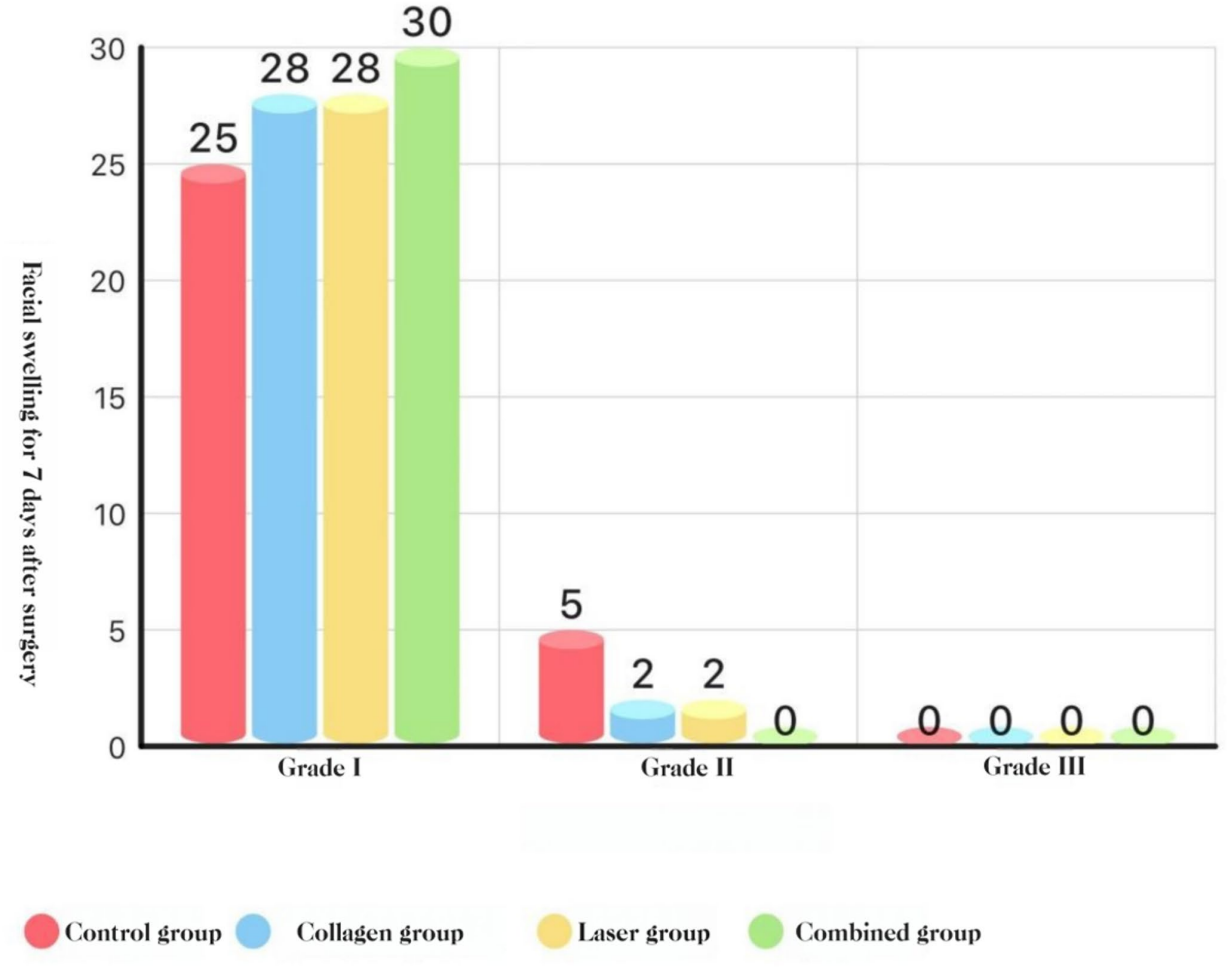
Distribution of facial swelling at POD 7 (Marković–Todorović grades): histogram

POD 7: no group differences (*P* > 0.05).**See** Figs. 8,9, and 10.

**Trismus** (change in maximum interincisal distance).

POD 1: COL + LAS was lower than BCG, COL, and LAS (*P* < 0.05).

POD 3: COL + LAS and LAS were lower than COL and BCG (*P* < 0.05).

**Fig. 11:**
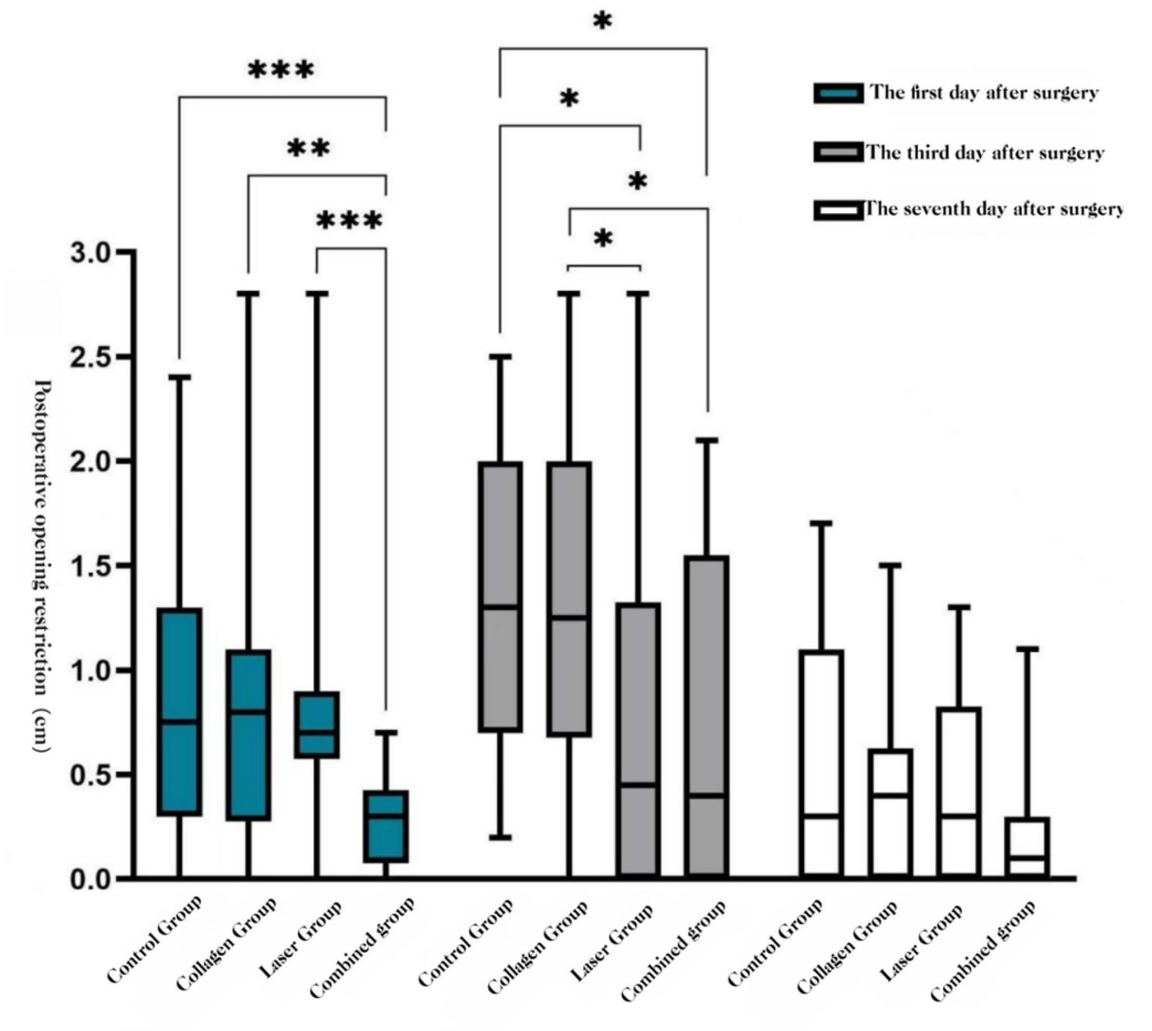
Trismus: box-and-whisker plots by group (POD 1, 3, 7)

**Fig. 12:**
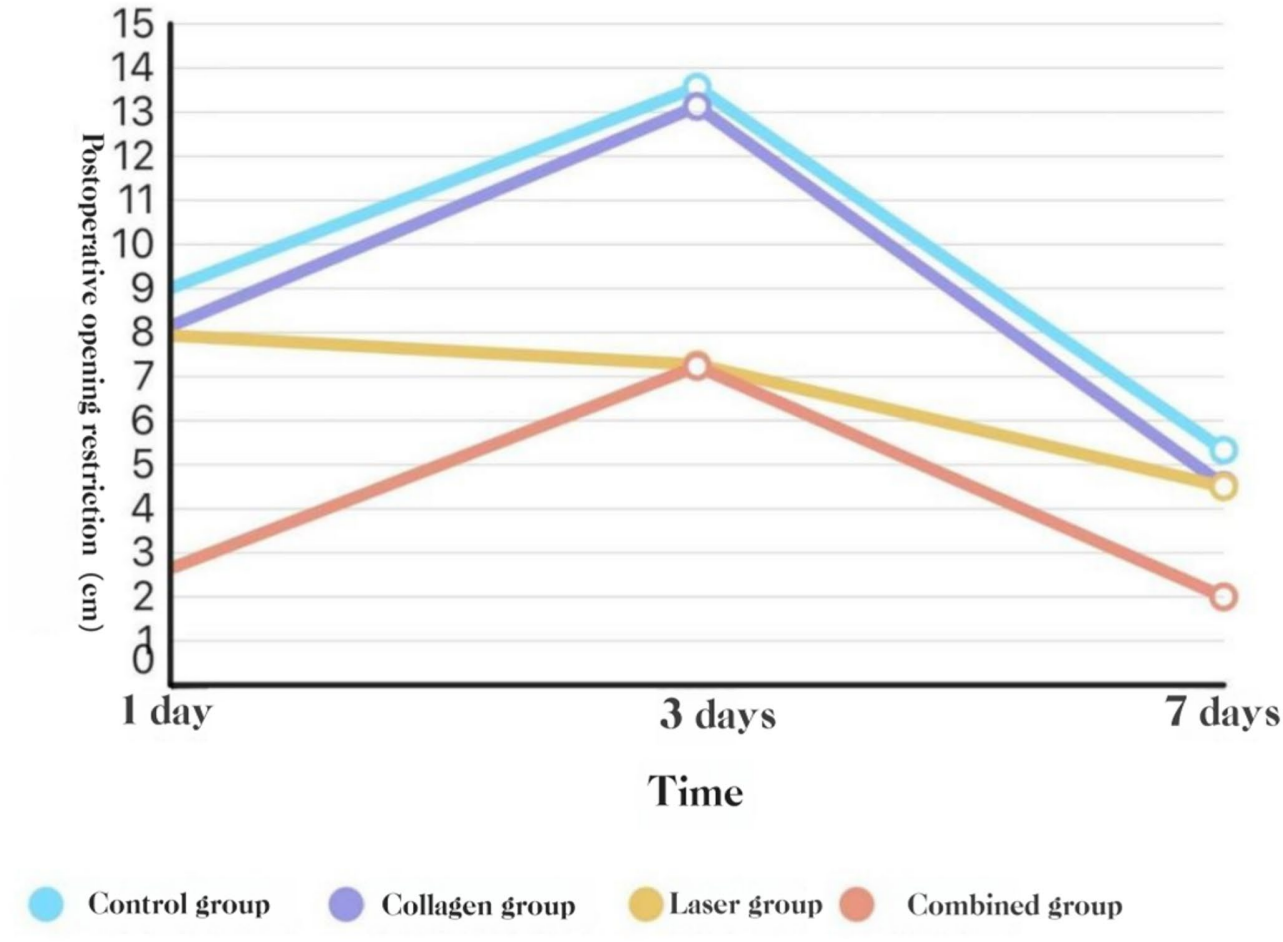
Trismus over time by group: mean ΔMID (POD 1, 3, 7

POD 7: no group differences (*P* > 0.05).See Figs. 11 and 12.

### Early mucosal healing (three-grade rubric)

**Fig. 13:**
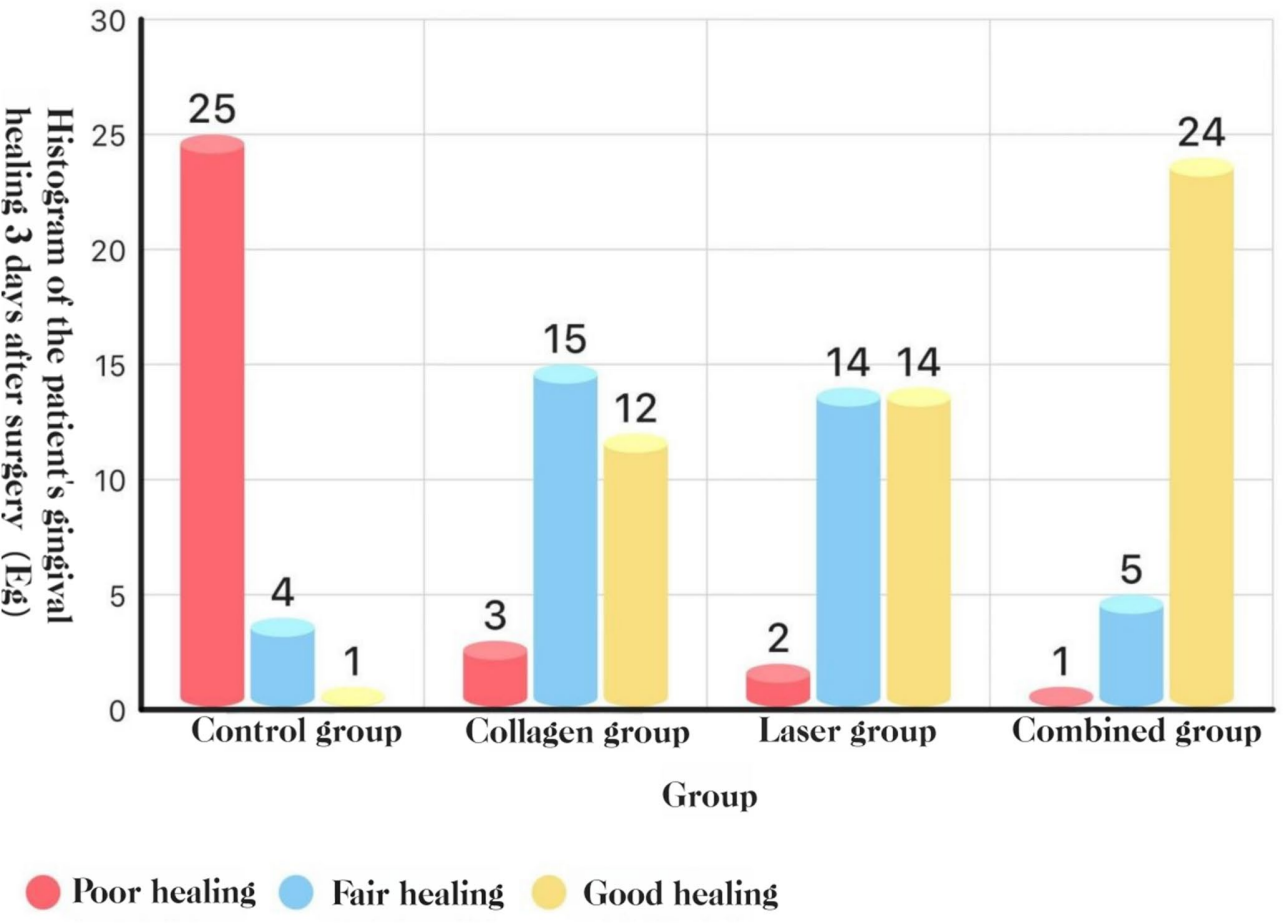
Distribution of mucosal healing grades at POD 3: histogram

**Fig. 14:**
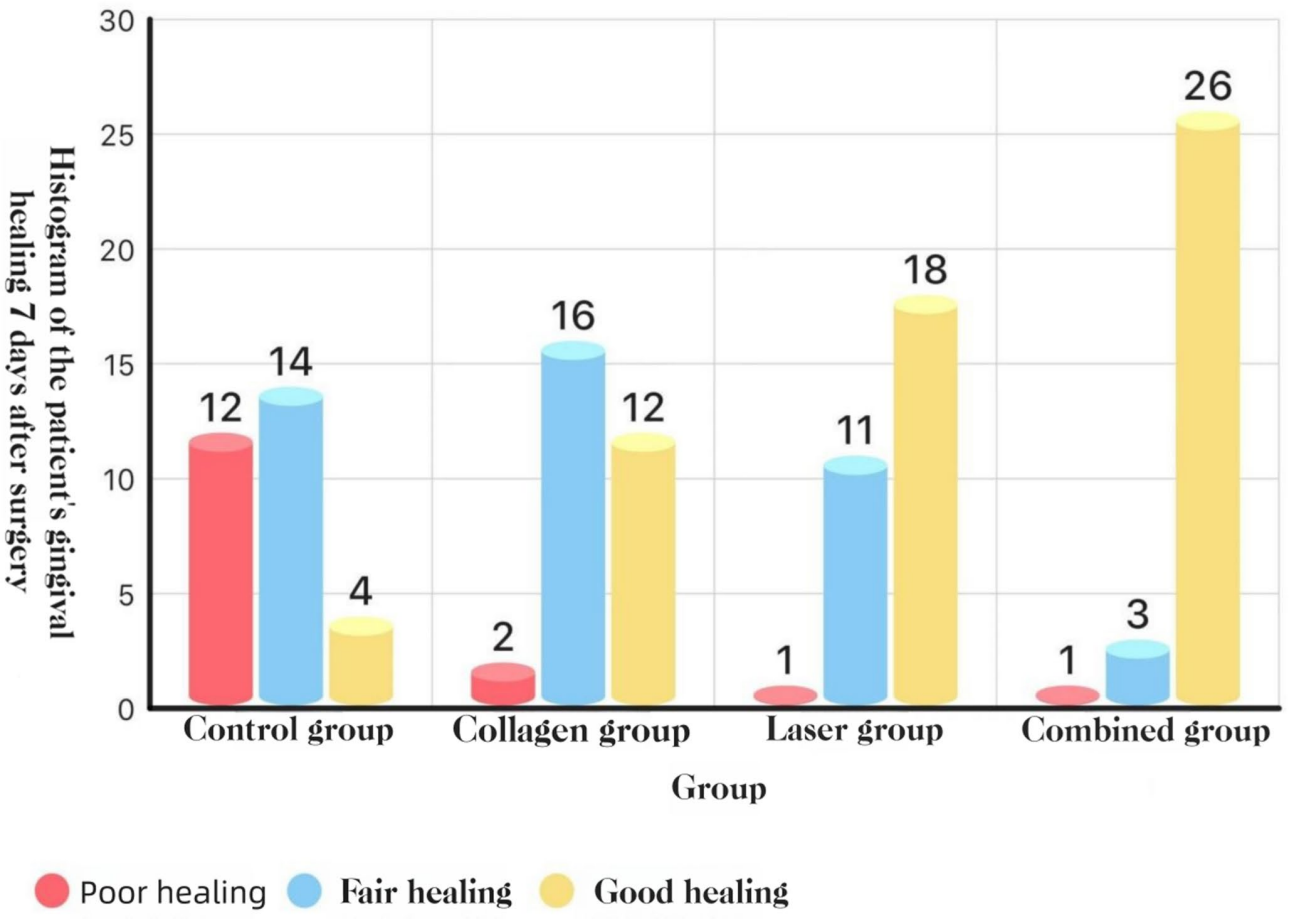
Distribution of mucosal healing grades at POD 7: histogram

At POD 3 and POD 7, COL + LAS achieved better healing than all other groups, and COL and LAS each outperformed BCG (all *P* < 0.05).See Figs. 13 and 14.

### Secondary endpoints

#### Bleeding at 30 min (three-level scale)

**Fig. 15:**
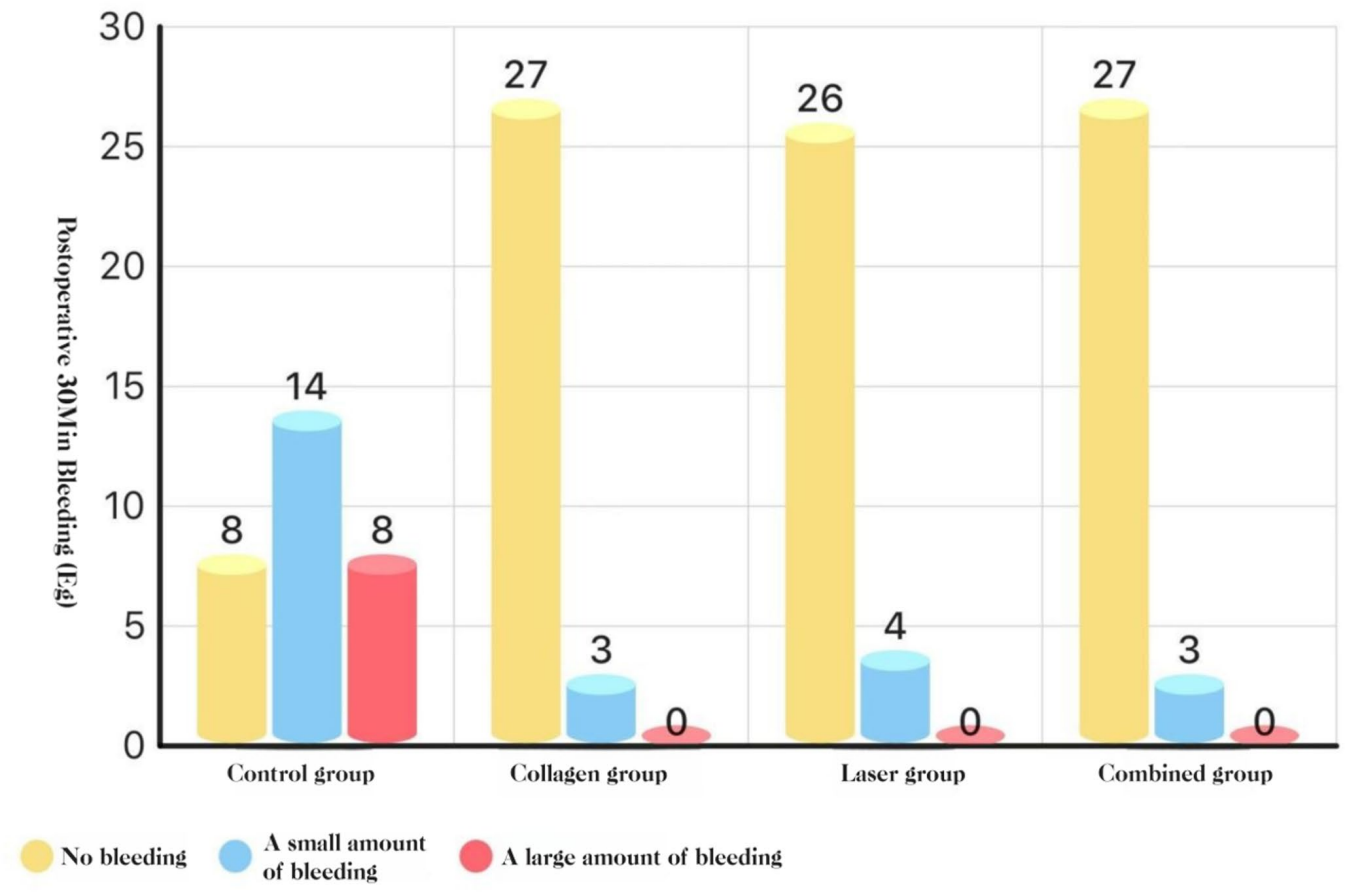
Distribution of bleeding grades at 30 minutes post-extraction: histogram

All active arms (COL, LAS, COL + LAS) were lower than BCG (*P* < 0.05), with no differences among the active arms (*P* > 0.05).**See** Fig. 15.

#### Cutaneous induration and ecchymosis (POD 7)

**Induration**: COL + LAS was lower than the other three groups, and COL and LAS were each lower than BCG (all *P* < 0.05).

**Fig. 16:**
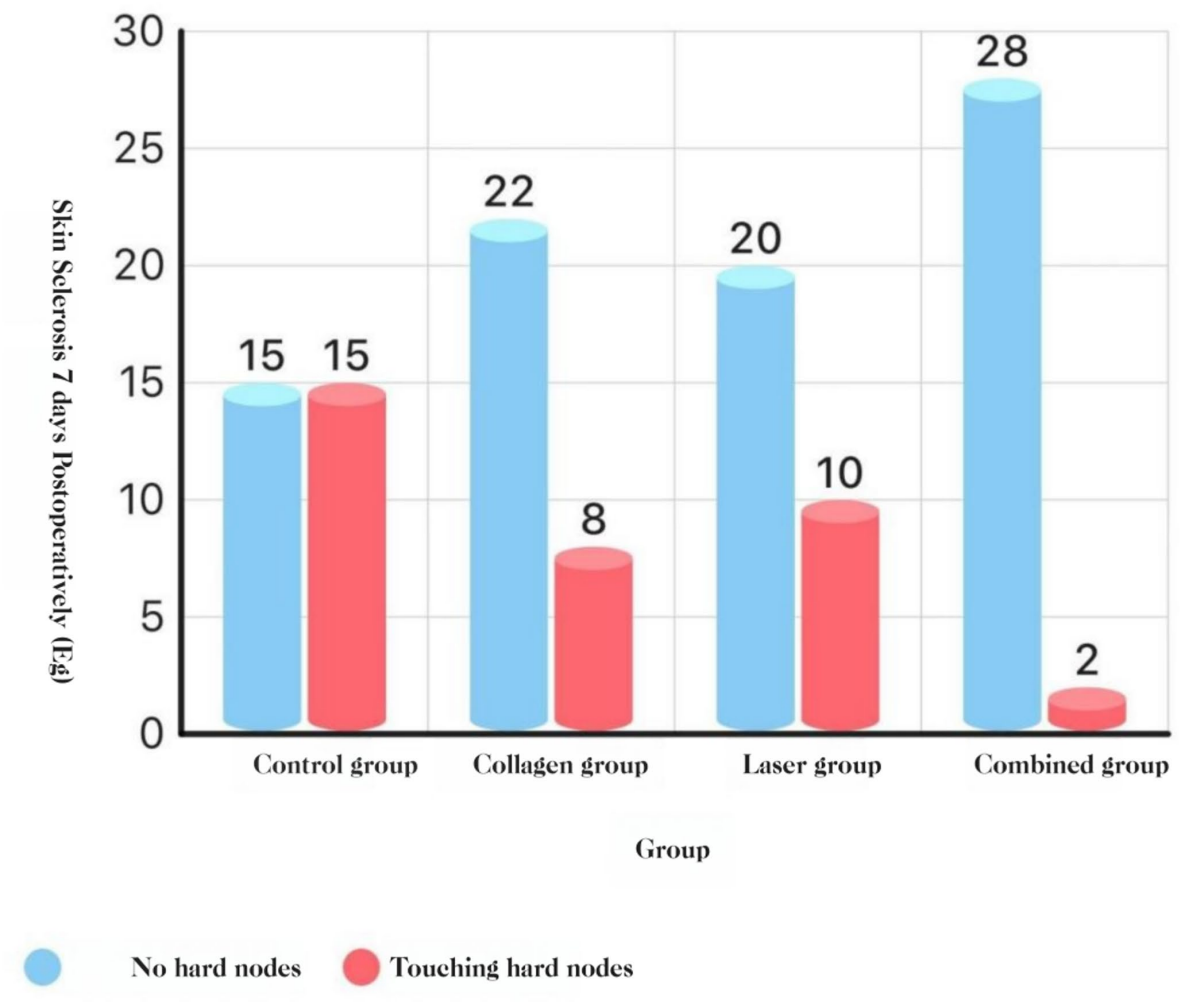
Cutaneous induration at POD 7

**Fig. 17:**
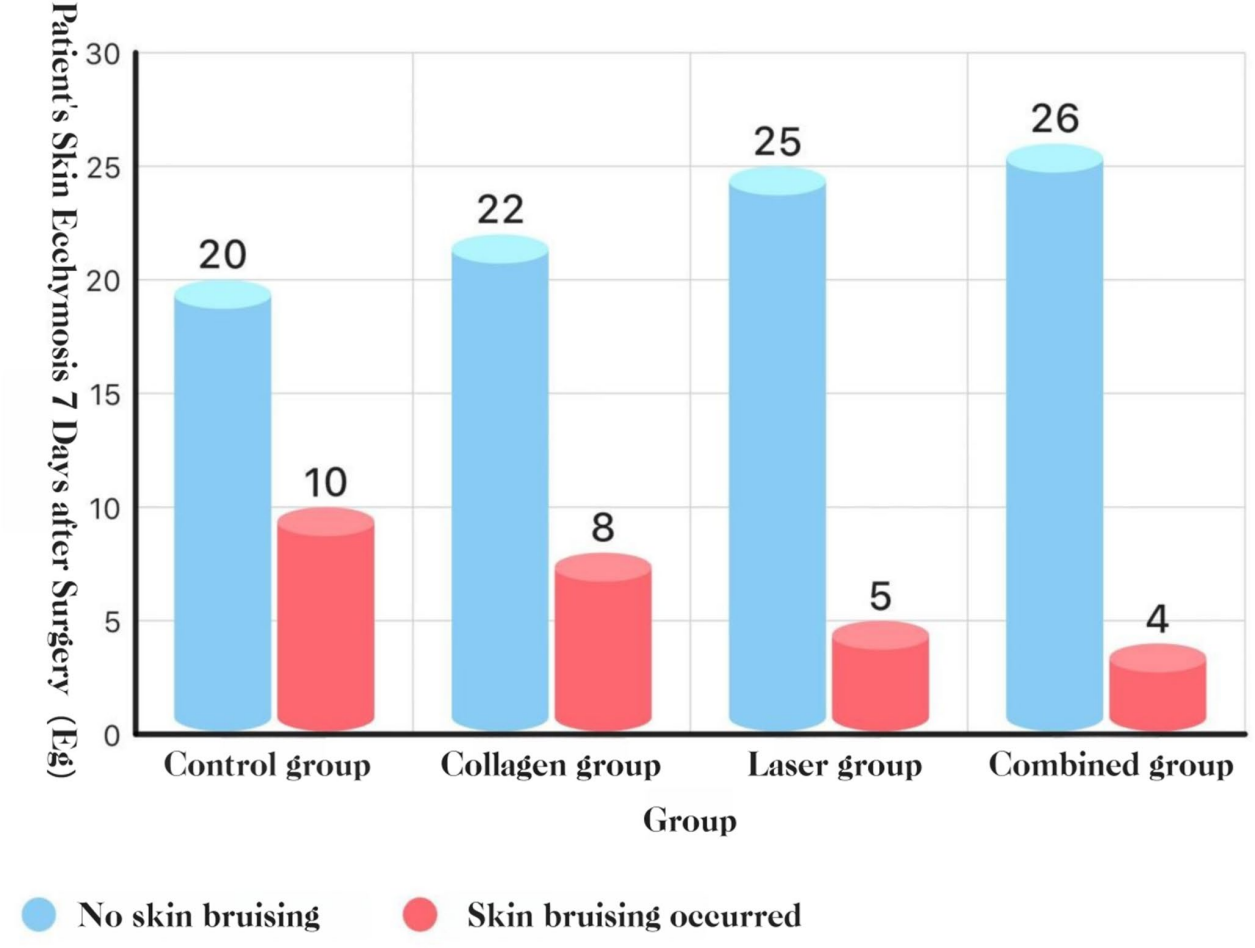
Cutaneous ecchymosis at POD 7

**Ecchymosis**: no group differences (*P* > 0.05).**See** Fig. 16, and 17.

### Alveolar osteitis

None occurred in any group.

### Local cytokines (socket exudate, ELISA)

**IL-1β.** POD 3: COL + LAS was lower than BCG and LAS; COL was lower than BCG (^*P*^<0.05). POD 7: COL + LAS was lower than all other groups, and COL and LAS were each lower than BCG (*P* < 0.05).

**TNF-α.** POD 3: COL + LAS was lower than the other three groups (*P* < 0.05). POD 7: COL + LAS was lower than all other groups, and COL and LAS were each lower than BCG (*P* < 0.05).

**Fig. 18:**
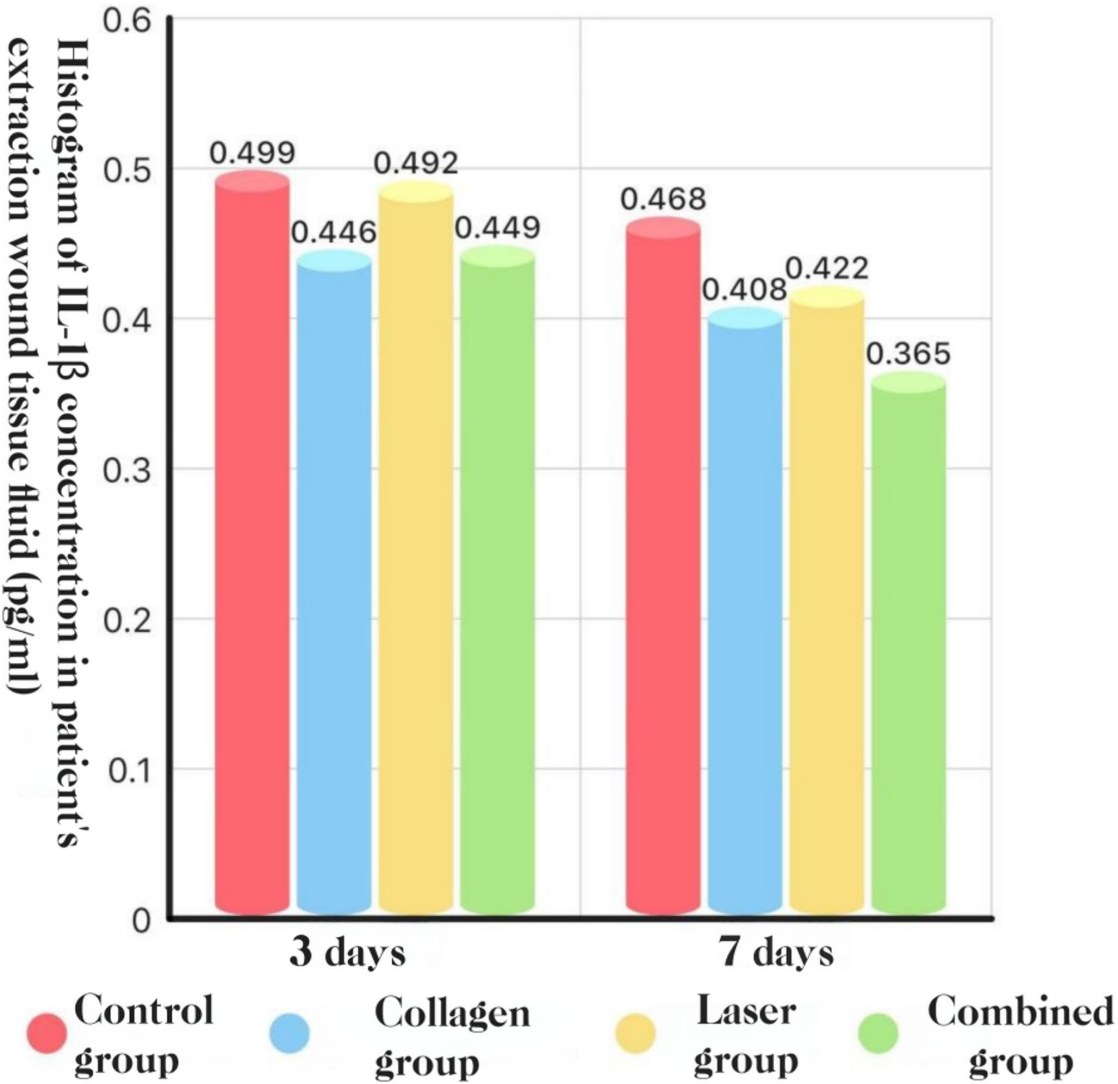
IL-1β concentration in socket exudate at POD 3 and POD 7: histogram

**Fig. 19:**
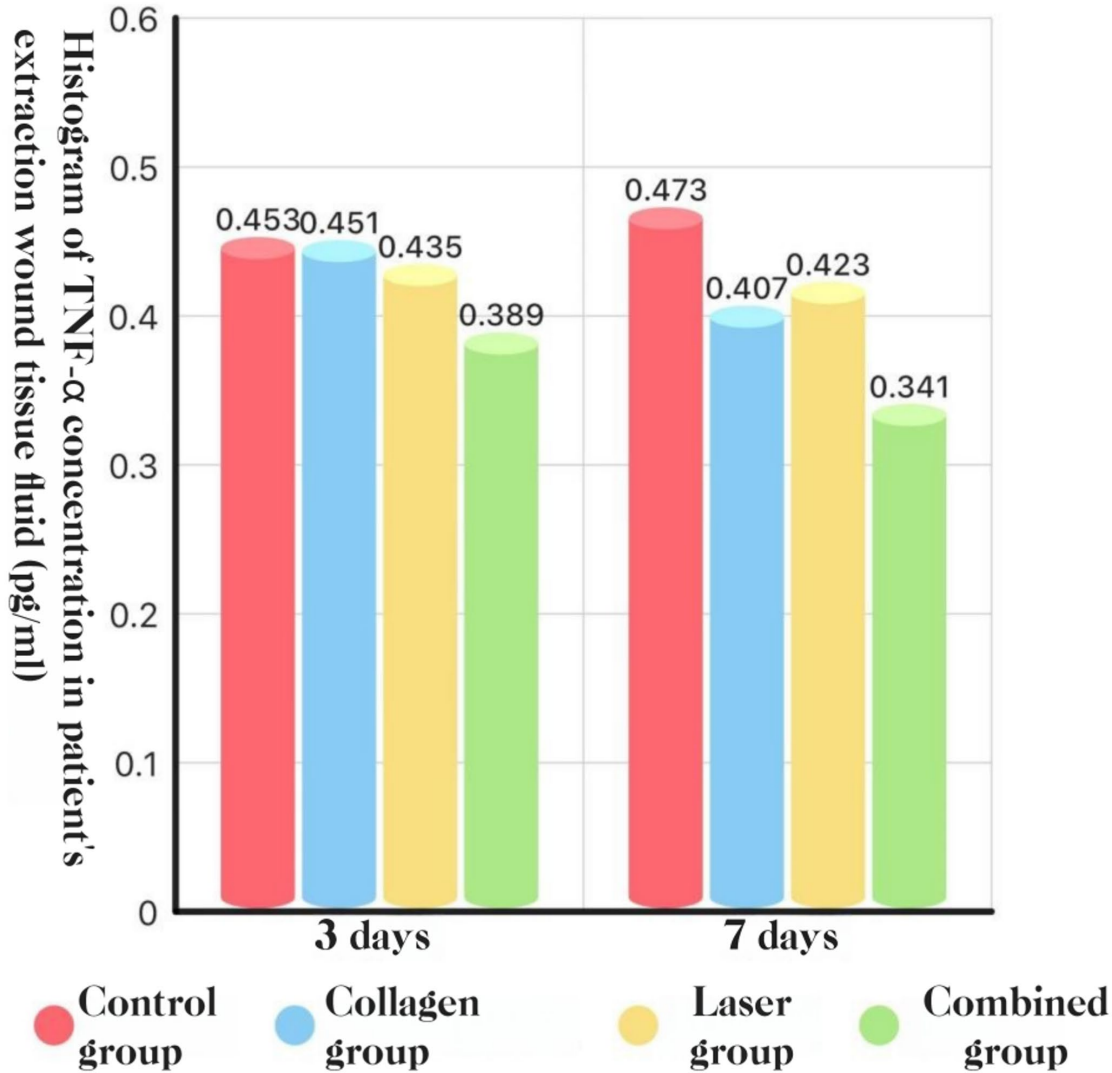
TNF-α concentration in socket exudate at POD 3 and POD 7: histogram

**Fig. 20:**
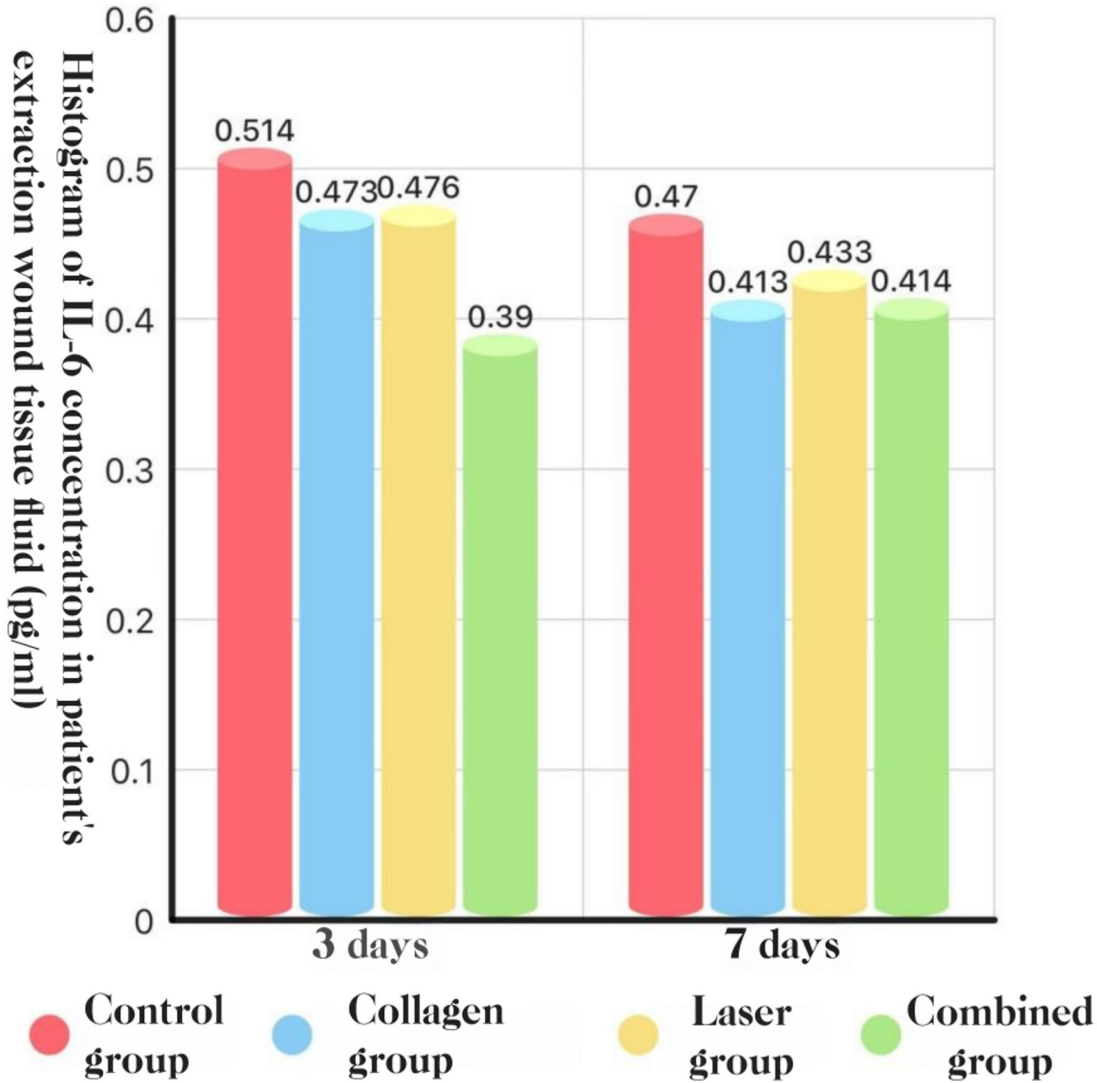
IL-6 concentration in socket exudate at POD 3 and POD 7: histogram

**IL-6.** POD 3: COL + LAS was lower than all other groups, and COL and LAS were each lower than BCG (*P* < 0.05). POD 7: COL, LAS, and COL + LAS were all lower than BCG (*P* < 0.05), with no differences among the active arms (*P* > 0.05).**See** Fig. 18, 19 and 20.

### COL + LAS: postoperative facial swelling and mucosal healing

(Figure 21.)

**Fig. 21:**
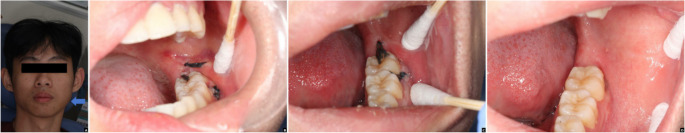
A, almost no obvious facial swelling at postoperative day 3; B, mucosal healing at postoperative day 3; C, mucosal healing at postoperative day 7; D, mucosal healing at postoperative week 3

## Discussion

### Observation of postoperative complications in cases treated with Er: YAG plus Nd: YAG lasers assisted by medical collagen

#### Pain

Our results indicate that both medical collagen alone and the combination of Er: YAG and Nd: YAG lasers reduce postoperative pain after extraction of impacted teeth, with the combined regimen providing superior analgesia. A meta-analysis of laser-assisted third-molar surgery reported that Er: YAG use improves postoperative pain [24], consistent with our findings. Because of its tissue penetration, the Nd: YAG laser can induce coagulation of cells—including neural elements—thereby decreasing nociceptor sensitivity and contributing to hemostasis and analgesia [20]. Bai Xijing and colleagues [25] similarly observed that application of medical collagen after tooth extraction reduces postoperative pain. In a split-mouth study of impacted mandibular third-molar removal, placement of a resorbable collagen sponge resulted in a significantly lower visual analogue scale (VAS) score at 1 week on the collagen side than on the control side (*P* < 0.05). Early postoperative facial swelling (weeks 1–2) was also reduced on the collagen side, although differences were no longer evident by week 14. These findings suggest that collagen sponge placement may lessen early postoperative complications [8], in line with our study.

#### Bleeding

At 30 min after extraction of impacted teeth, blood loss was lower in the collagen, laser, and combined-treatment groups than in the blank control group. This reduction is biologically plausible: the Nd: YAG laser, with relatively deep tissue penetration and melanin absorption, induces coagulation within the tissue and thereby promotes hemostasis [20]. Consistently, in a murine model, Nd: YAG irradiation produced instantaneous thrombus formation in dorsal skin, confirming its hemostatic action [26]. Collagen also contributes to hemostasis by swelling upon water absorption, stabilizing the clot and supporting wound closure [25]. Clinically, immediate application of a collagen-based cylindrical hemostatic dressing (ETIK COLLAGENE) after tooth extraction shortened mean bleeding time and reduced postoperative adverse events compared with an 8-minute delayed application [27]. Together, these data align with our findings of reduced early postoperative bleeding in the intervention groups.

#### Swelling

Our results show that both medical collagen alone and the combined use of Er: YAG and Nd: YAG lasers reduce postoperative swelling after removal of impacted third molars, with the combination providing the greatest benefit. Giovannacci et al. [15] reported less postoperative swelling with Er: YAG-assisted extraction compared with conventional turbine techniques, consistent with our findings. In a comparative study, postoperative Nd: YAG irradiation achieved swelling control comparable to intramuscular nonsteroidal anti-inflammatory drugs (NSAIDs), suggesting that Nd: YAG may limit reliance on NSAIDs without compromising efficacy. Jiao Kun and colleagues [28] likewise found that application of medical collagen after extraction attenuated postoperative swelling, in agreement with the present study.

#### Trismus

The combined use of Er: YAG and Nd: YAG lasers with adjunctive collagen reduced trismus on postoperative days 1 and 3. Trismus after extraction is largely driven by inflammation involving the masticatory muscles—the masseter, medial pterygoid, and temporalis—resulting in reactive spasm and limited mouth opening [5]. This observation is consistent with reports by Sales et al. [24] and Giovannacci [15]. Owing to its precise cutting and minimal collateral thermal injury, the Er: YAG laser can decrease surgical trauma during impacted-tooth surgery and thereby mitigate postoperative trismus. In addition, a study of non-surgical periodontal therapy showed that Er: YAG treatment improved probing depth and clinical attachment, findings that may translate to reduced postoperative trismus [29]. By contrast, placement of medical collagen into the socket after impacted-tooth extraction did not significantly affect trismus compared with blank controls, indicating that collagen alone does not alleviate postoperative limitation of mouth opening, in agreement with Jiao Kun et al. [28].

#### Cutaneous induration and ecchymosis

Post-extraction cutaneous induration and ecchymosis stem from two processes: (i) trauma-related bleeding with intratissue blood stasis and (ii) postoperative inflammation that increases capillary permeability, impairs lymphatic drainage, and sustains edema; when unresolved, these changes manifest as induration and bruising. Our results show that the combined-treatment group had fewer cases of cutaneous induration than either the collagen or laser group, and both had fewer cases than the blank control group; these differences were statistically significant. These findings suggest that collagen, Nd: YAG therapy, and their combination reduce post-extraction cutaneous induration after removal of impacted teeth. By contrast, the incidence of cutaneous ecchymosis did not differ significantly among the four groups; however, absolute counts favored the combination (2 cases) over the blank control (10 cases). Although not statistically significant, this trend may still be clinically relevant. The results are consistent with the hematoma outcomes reported by Giovannacci et al. [15]. Given the pathophysiology of hematoma and ecchymosis, minimizing bleeding remains a key preventive strategy; as discussed above, collagen, Er: YAG plus Nd: YAG therapy, or their combination can reduce bleeding and thereby lower the risk of cutaneous induration and ecchymosis.

#### Alveolar osteitis

No cases of alveolar osteitis occurred in any of the four groups. Nd: YAG laser therapy may facilitate post-extraction wound healing in patients at high risk for jaw osteomyelitis, such as those receiving long-term bisphosphonates [30]. Nd: YAG irradiation also promotes osteogenesis by upregulating BMP-2 expression in preosteoblasts, thereby inducing cellular proliferation and differentiation; when applied within periodontal pockets, it exerts additional antibacterial effects [31]. Bai Xijing et al. [25] reported that medical collagen enhances hemostasis after removal of impacted teeth, reduces postoperative pain, and lowers the incidence of alveolar osteitis. Similarly, Yuan Xuguang et al. [32] found in a large comparative cohort that collagen substantially decreases the risk of alveolar osteitis. In light of our results, plausible explanations include: (i) advances toward minimally invasive extraction—using high-speed contra-angle electric handpieces and piezosurgery rather than traditional techniques—which markedly reduce the risk of alveolar osteitis; and (ii) standardized operative protocols, strong patient adherence, and adjuvant use of antibiotics and mouth rinses.

#### Mucosal healing

Postoperative assessments showed higher proportions of “good” mucosal healing on days 3 and 7 in the combination, collagen, and laser groups than in the blank control group, with statistically significant differences. Moreover, the combination group outperformed both single-modality groups, indicating that the combined regimen better supports mucosal healing after removal of impacted teeth. Mechanistically, Er: YAG irradiation promotes type I collagen formation and osteogenesis and, together with the biostimulatory and antimicrobial actions of laser therapy, facilitates wound repair [15, 16]. Nd: YAG irradiation further inactivates bacteria and virulence factors such as endotoxin, thereby reducing postoperative infection and promoting soft-tissue healing [19]. In patients on long-term bisphosphonates, Nd: YAG use enhanced gingival and bone healing after extraction [30]. Consistently, Ma Yang et al. [33] reported that medical collagen promotes soft-tissue repair. As a biocompatible scaffold, collagen— the principal organic component of bone—supports bone formation and mineralization. Collagen sponges promote post-extraction bone healing through their porous architecture, which favors cell seeding and osteoblastic differentiation [34]. In impacted third-molar surgery, collagen sponges are placed in extraction sockets to prevent soft-tissue collapse, reduce postoperative complications, and promote early healing of soft tissues and periodontal defects [35]. During alveolar-socket healing, collagen induces a vascular network rich in H-type vessels, fostering new bone formation critical to early socket repair [36]. In summary, collagen sponges and membranes enhance bone repair and reduce complications, making them key materials for alveolar-ridge preservation; future studies should clarify their long-term effects on bone healing and periodontal regeneration.

#### Pro-inflammatory cytokines: IL-1β, IL-6, and TNF-α

Interleukin-1β (IL-1β) and interleukin-6 (IL-6) are chemotactic cytokines that amplify inflammation, activate neutrophils and macrophages, promote alveolar bone resorption and febrile responses, and play key roles in infection and tissue injury [37]. Tumor necrosis factor-α (TNF-α) is a pivotal early mediator that recruits inflammatory cells to sites of infection; it activates neutrophils and lymphocytes, modulates tissue metabolism, and stimulates the release of other cytokines (e.g., IL-6 and IL-1β), acting synergistically in pain signaling [38].

In this study, local cytokine production was evident after extraction of impacted teeth; however, combining Er: YAG and Nd: YAG lasers with adjunctive collagen reduced IL-1β, IL-6, and TNF-α levels at the surgical site, which may partially account for the concomitant reduction in complications. Guo Tao et al. [39] reported that Er: YAG laser debridement of inflammatory granulation tissue accelerated healing and decreased postoperative pain and inflammation, with lower TNF-α, IL-1β, and IL-6 concentrations in socket exudate than in conventional care—findings consistent with ours. Similarly, Bai Xijing et al. [25] observed that placement of collagen within the extraction socket lowered inflammatory cytokine levels, again aligning with the present results.

Overall, all three interventions—combination therapy, laser alone, and collagen alone—outperformed the blank control in mitigating postoperative complications. Among them, the combination therapy yielded the greatest reductions in pain, bleeding, facial swelling, trismus, and cutaneous induration after removal of impacted teeth, followed by the Er: YAG/Nd: YAG laser regimen and then collagen alone.

## Conclusion

The combined application of Er: YAG and Nd: YAG lasers with adjunctive collagen in impacted third-molar surgery reduces postoperative complications—including pain, bleeding, facial swelling, trismus, and cutaneous induration—facilitates socket healing, and improves postoperative quality of life, supporting broader clinical adoption. Moreover, this regimen lowers local levels of TNF-α, IL-1β, and IL-6 at the surgical site, offering clinically relevant evidence to inform practice.

## Supplementary Information

Below is the link to the electronic supplementary material.

Supplementary Material 1 (DOCX 1.17 MB)

Supplementary Material 2 (DOCX 1.17 MB)

## Data Availability

Additional data are available in the Supplementary Material.

## Declarations

### Clinical trial number

PID:248,812.
