# Supplementary material for: Er:YAG and Nd:YAG-based low-level laser therapy (LLLT) with medical collagen improve third-molar extraction wound healing: a randomized controlled trial
Source: Lasers Med Sci. 2025 Dec 8;40(1):512. doi: 10.1007/s10103-025-04763-7 (PMC12682916; doi:10.1007/s10103-025-04763-7)

### Supplementary material

**Appendix I Typical Cases**

**Typical Case Presentation (1)**

**Patient Name: Li XX；Patient Age: 25 years old ；Patient Gender: Male**

**I. Medical History Summary**

****1.** **Chief Complaint:** The patient presented with ‘recurrent swelling and pain in the left lower wisdom tooth for over a month.’**

****2. Current Medical History:** The patient began experiencing swelling and pain in the left lower wisdom tooth area one month ago. The pain fluctuates in intensity, worsening during meals. Symptoms temporarily improve after self-administering anti-inflammatory medication but do not resolve completely.**

****3. Past Medical History:** Denies systemic diseases; no history of drug allergies.**

****4. Family History:** No significant family medical history.**

****5. Personal history:** No history of smoking or alcohol consumption; oral hygiene is generally adequate.**

****II. Clinical examination****

**1. Specialised examination:**

**Extra-oral Examination:**

****Facial appearance and colour:** No obvious asymmetry in facial appearance; skin colour is normal, with no jaundice or pigmentation.**

****Swelling and tenderness:** No significant swelling in the left mandibular angle region; mild tenderness on palpation, with no fluctuation or crepitus.**

****Facial and jaw bones:** No obvious abnormalities in bone structure, no step-off or depression, no bone crepitus or abnormal mobility.**

****Temporomandibular joint:** No pain during jaw opening and closing movements, no clicking sounds, normal mouth opening range, no obvious abnormal sounds detected.**

****Facial and neck lymph nodes:** No obvious enlargement of submandibular or neck lymph nodes, no tenderness.**

**Intra-oral Examination**:****

****Oral hygiene:** Good oral hygiene, no large tartar deposits.**

****Gums:** No redness or swelling around the left lower third molar, no gum recession, normal width of keratinised gums.**

****Teeth:** The left lower third molar has not erupted, covered by surrounding gums, food debris can be detected, no obvious pus discharge.**

****Mobility:** No significant mobility was noted in the teeth surrounding the left lower third molar.**

****Probing Bleeding and Depth:** No bleeding was observed after probing the gingiva surrounding the left lower third molar, with a probing depth of approximately 2 mm.**

**Other Examinations:**

**Speech: The patient's speech is clear, with no obvious impairments.**

**Salivary Glands: Saliva secretion in the oral cavity is normal, with no reduction or increase, and no redness or pus discharge at the duct openings.**

**III.Panoramic X-ray**

**The lower left third molar is horizontally impacted, with the crown in contact with the neck of the second molar. The periodontal ligament is widened, and no obvious resorption of the adjacent alveolar bone is observed. The apex is close to the inferior alveolar nerve canal.**


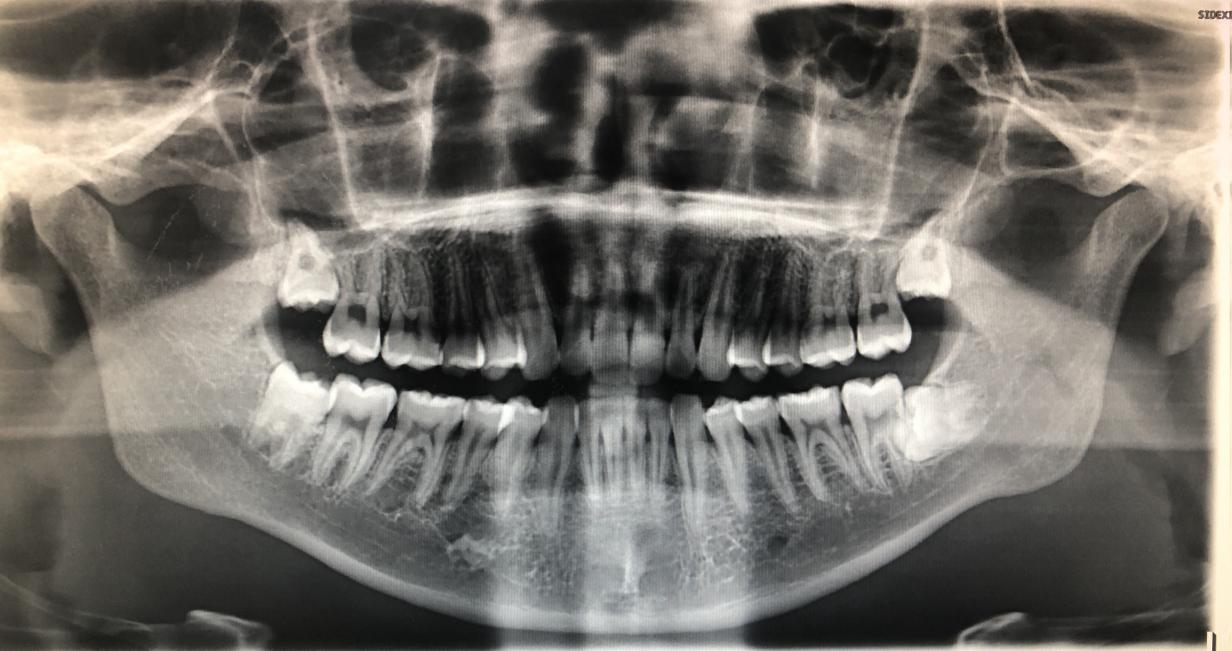


**IV.Preliminary diagnosis**

**38 impacted tooth (median level impaction).**

**V. Treatment Plan**

**1. Treatment Plan: Use Er:YAG laser combined with Nd:YAG laser and collagen protein for minimally invasive extraction of the impacted lower left tooth.**

**2. Preoperative Preparation: The patient has completed preoperative blood routine and coagulation function tests, with normal results and no contraindications for surgery.**

**VI.Surgical Procedure**

**1. Anaesthesia Method:** Block anaesthesia is administered to the inferior alveolar nerve, lingual nerve, and buccal nerve to ensure patient comfort and safety during the procedure.

**2.Surgical procedure**：****

1. First, an Er:YAG laser (wavelength 2,940 nm) is used for gingival incision and separation of the periodontal ligament. The Er:YAG laser, due to its high absorption of water and hydroxyapatite, effectively minimises thermal damage to surrounding healthy tissues while achieving precise cutting of soft tissues.。
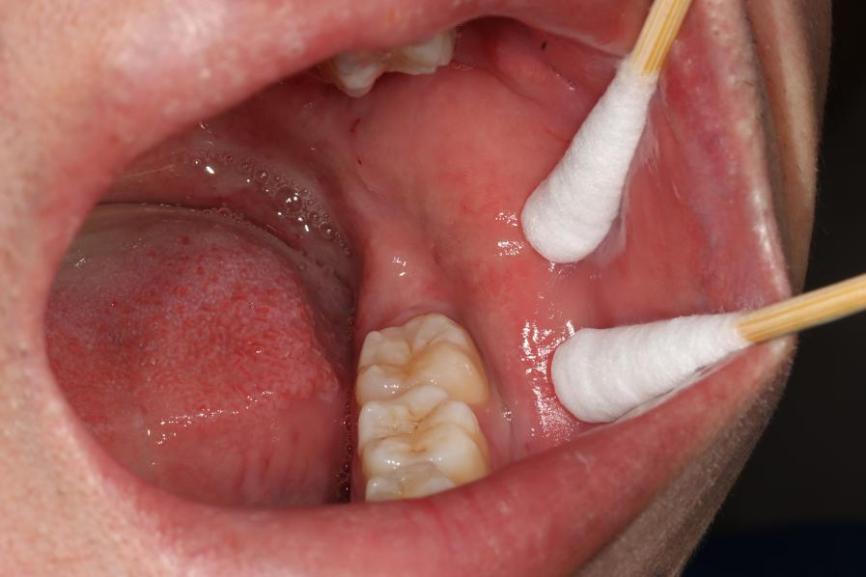

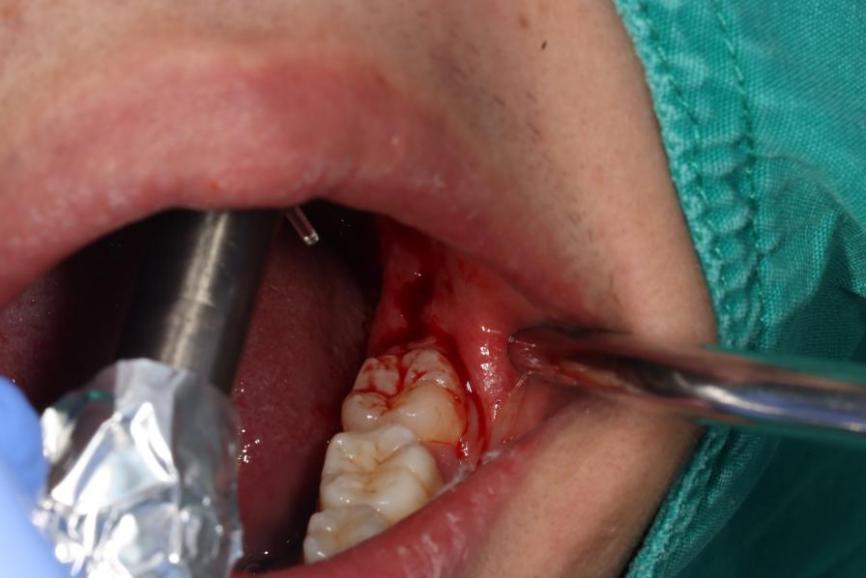

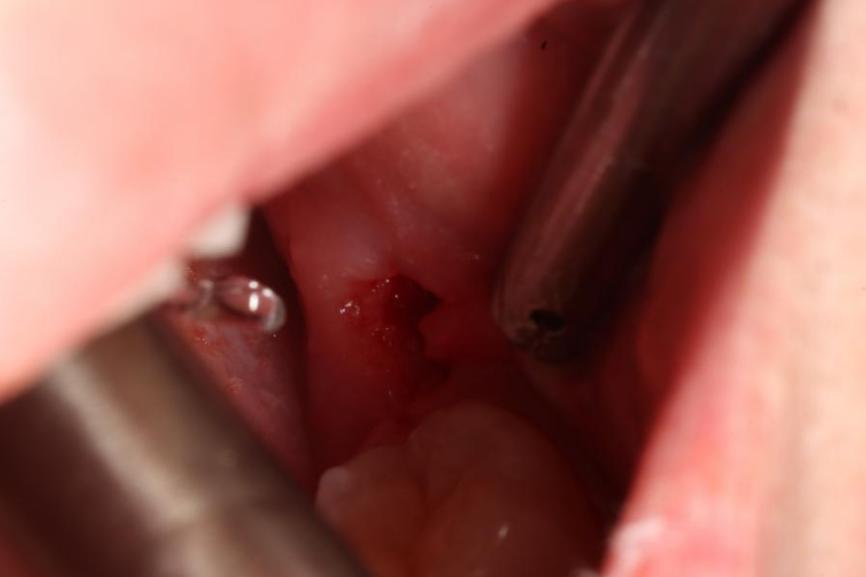

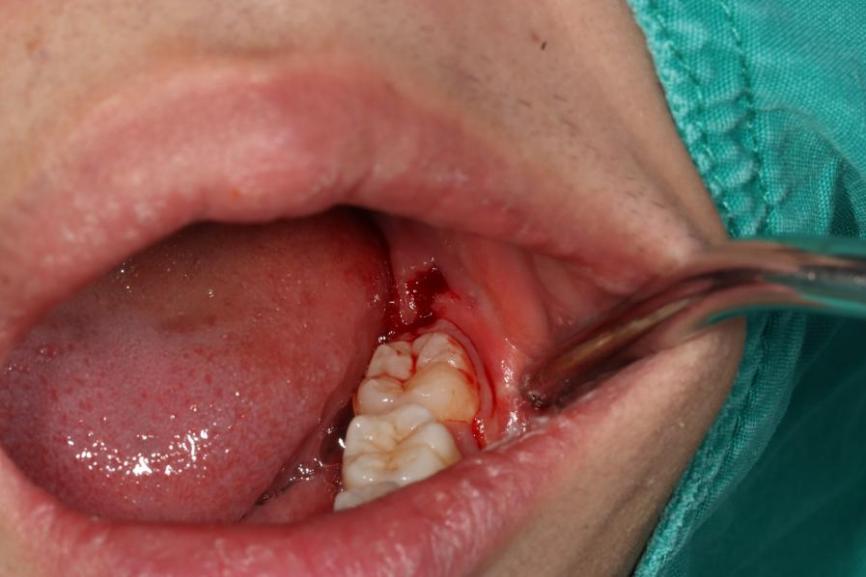


2. Minimally invasive tooth extraction procedure: After incising and lifting the flap, use an ultrasonic bone cutter to remove bone and create space, and then use a high-speed handpiece to separate and extract the affected tooth.。
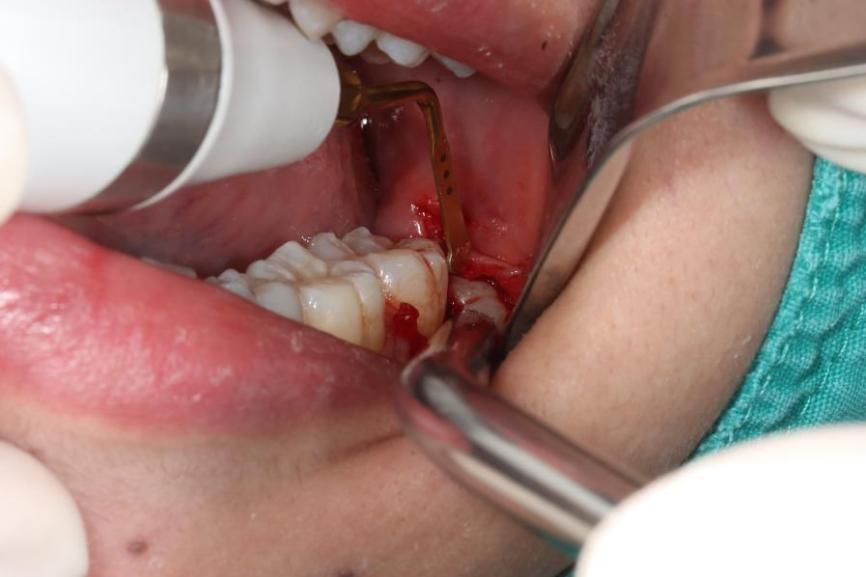

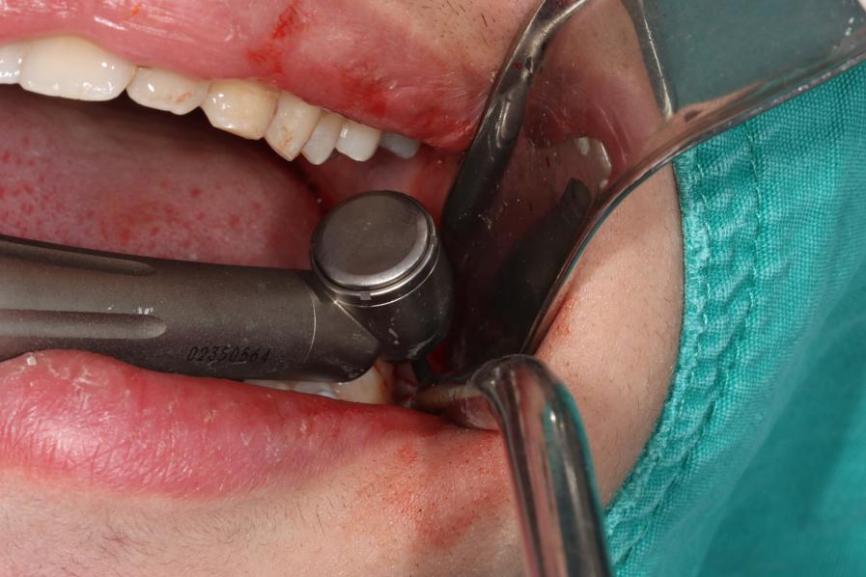


3. Er:YAG laser (wavelength 2,940 nm) is used again to remove granulation tissue, followed by Nd:YAG laser (wavelength 1,064 nm) for local soft tissue physiotherapy. Nd:YAG laser has good tissue penetration and is effectively absorbed by haemoglobin and melanin, making it suitable for soft tissue ablation and haemostasis.


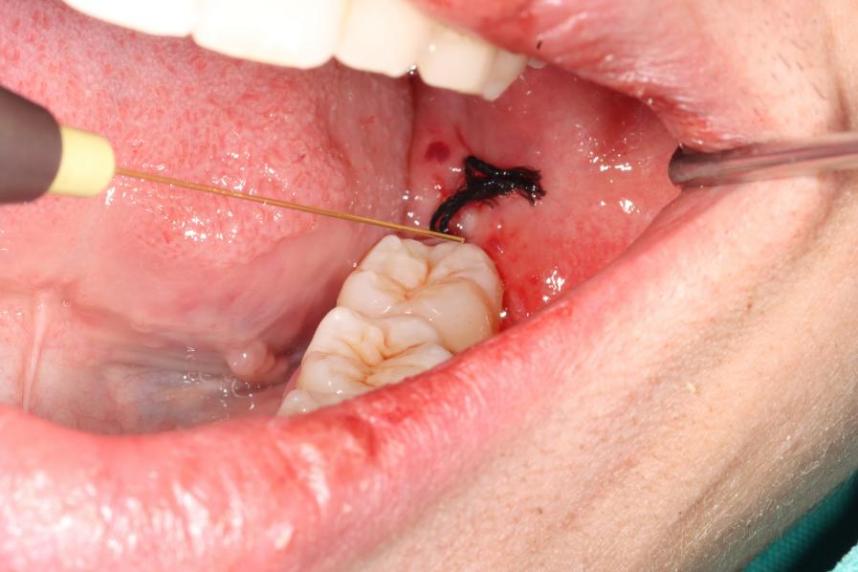


（Surgical site therapy）


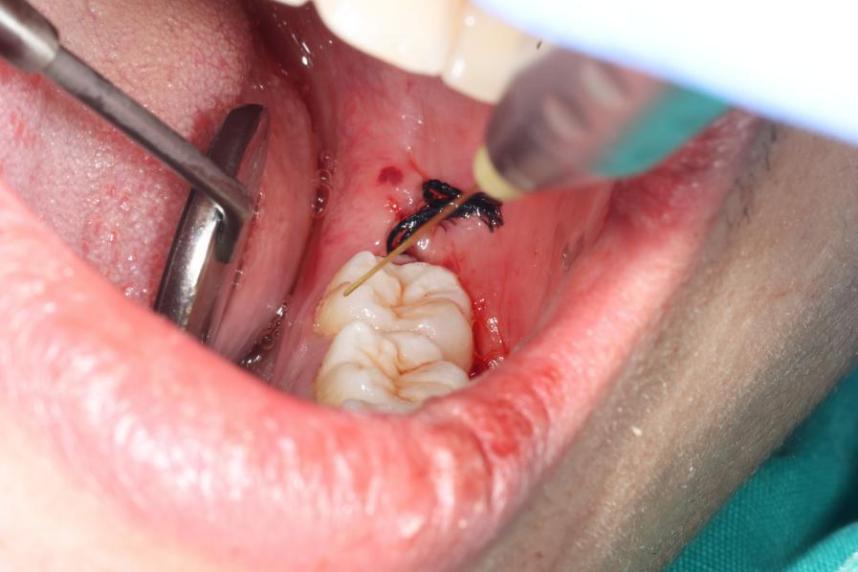


（Pharyngeal vestibule physiotherapy）


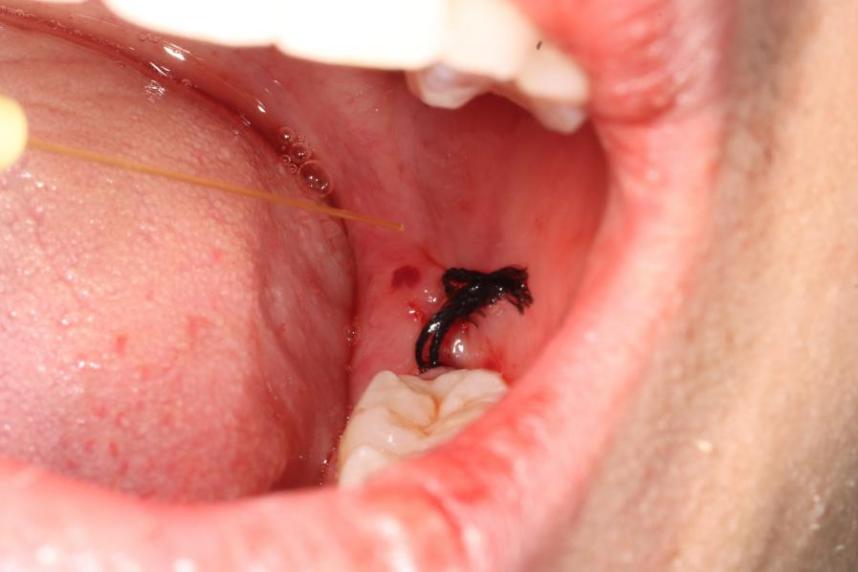


（Wing-under jaw area physiotherapy）


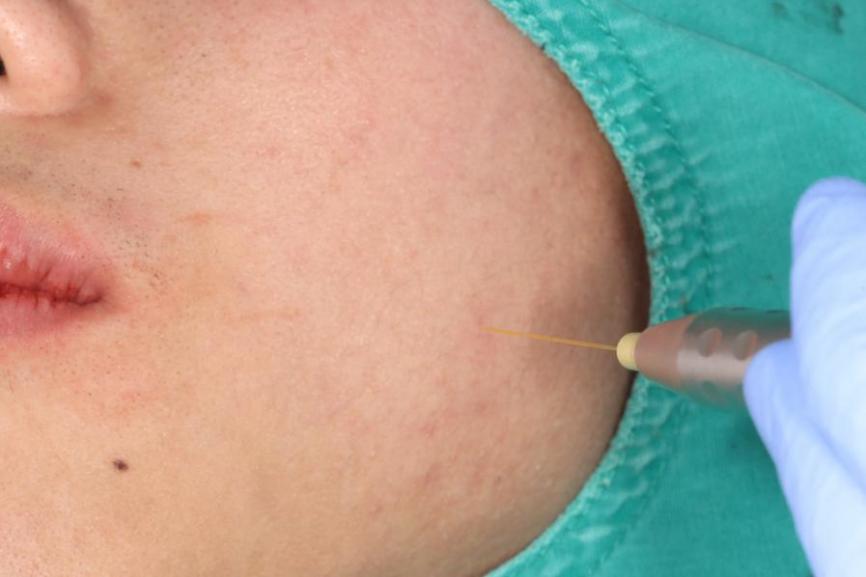


（Massage therapy for the masseter muscle area）

4.Finally, collagen is filled into the wound.。

5.Under the action of laser, the impacted tooth was successfully extracted, while reducing bleeding during surgery and post-operative swelling.

**VII.postoperative management**

**1.Postoperative instructions: Due to the minimally invasive nature of laser surgery, postoperative pain and swelling are mild, and patients recover more quickly. Antibiotics are administered to prevent infection, and pain medication is given to relieve postoperative pain. Patients are instructed to avoid hot foods for 24 hours after surgery and to maintain oral hygiene.**

**2. Follow-up plan: Follow-up examinations are scheduled for 3 days and 7 days after surgery to assess healing.**

**VIII.Postoperative results**

**1. Wound healing: During follow-up examinations on days 3 and 7 post-surgery, the wound healing was good, with no signs of infection. The patient reported mild post-operative pain and expressed satisfaction with the surgical outcome.**

**2. Swelling and limited mouth opening: On day 3 post-surgery, the patient experienced mild swelling in the cheek area, but the swelling was not significant and did not cause discomfort. The patient's mouth opening has returned to normal, with no signs of limited mouth opening.**

**3 days after surgery**:


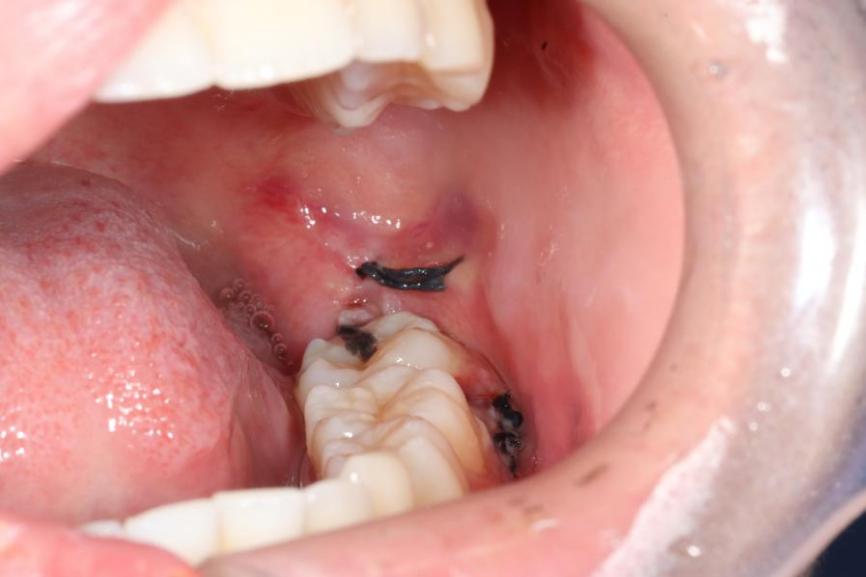


Er::YAG, Nd:YAG

Laser combined with collagen

VS


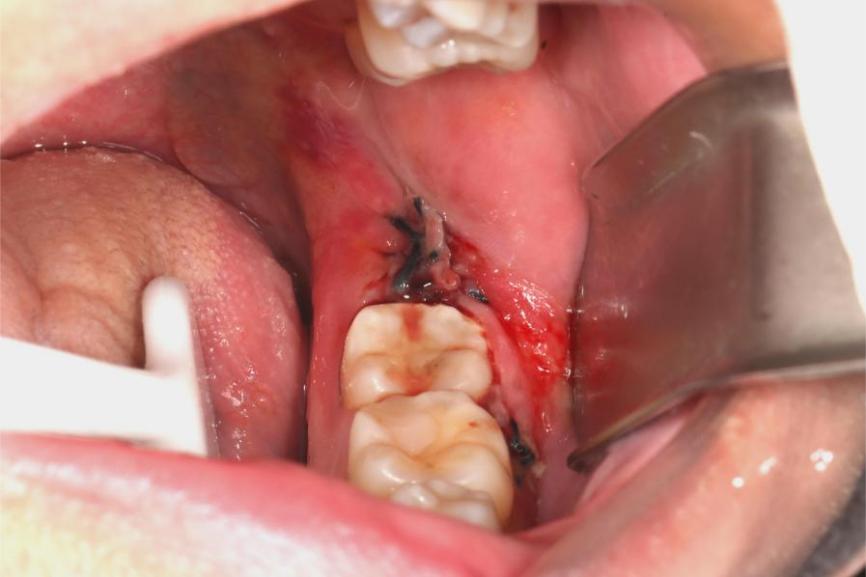


Blank control group

（The comparative results at 3 days post-surgery showed that the treatment regimen combining Er:YAG laser (wavelength 2,940 nm) with Nd:YAG laser (wavelength 1,064 nm) and collagen resulted in healthier pink-coloured gingival tissue at the surgical site, milder redness and swelling, and better healing outcomes compared to traditional treatment methods. The advantage of this therapy lies in the Er:YAG laser's high absorption of water and hydroxyapatite, enabling precise soft tissue cutting while minimising thermal damage to surrounding healthy tissues. The Nd:YAG laser, on the other hand, has excellent tissue penetration and is effectively absorbed by haemoglobin and melanin, making it suitable for soft tissue ablation and haemostasis. The use of collagen helps promote wound healing and reduce post-operative bruising, thereby accelerating the recovery process and minimising post-operative discomfort and complications.）


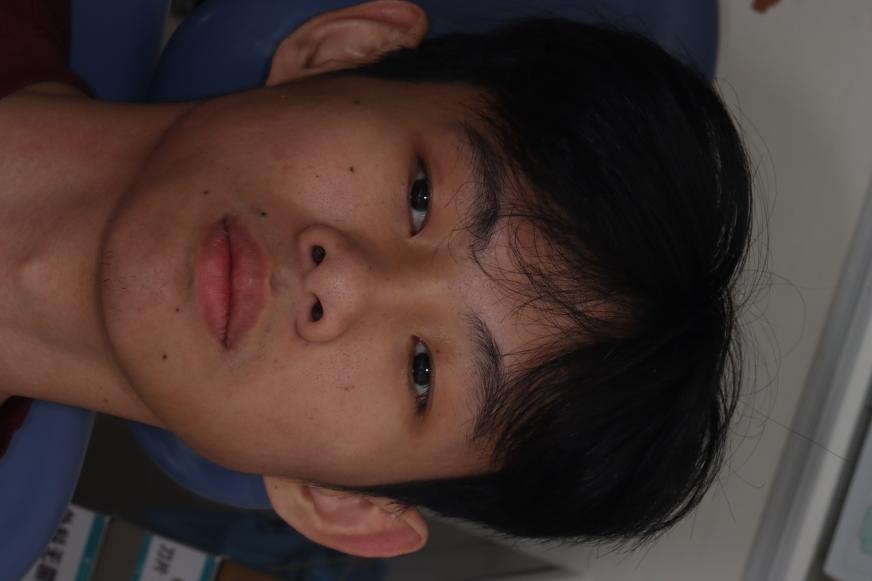


（3 days after surgery: The patient has slight swelling in the cheek area, but the swelling is not noticeable.)

**7 days after surgery：**


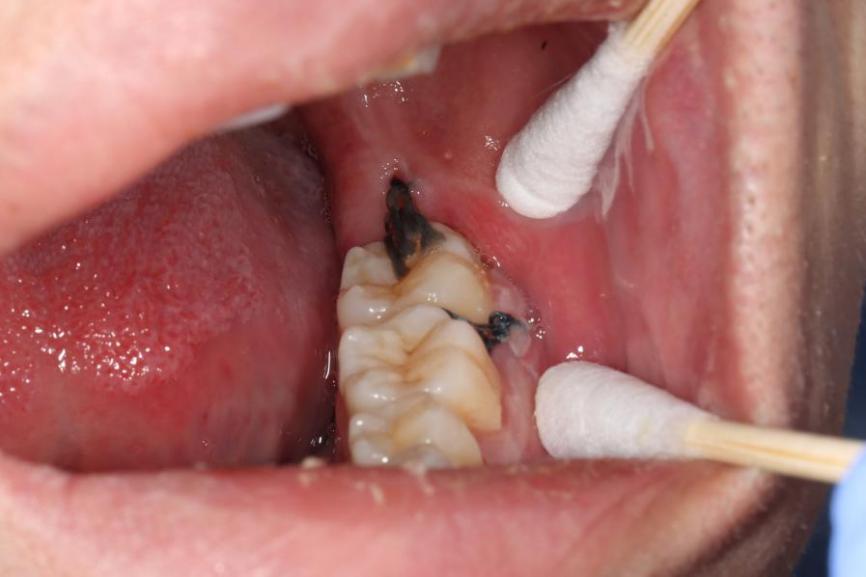


**3 weeks after surgery：**


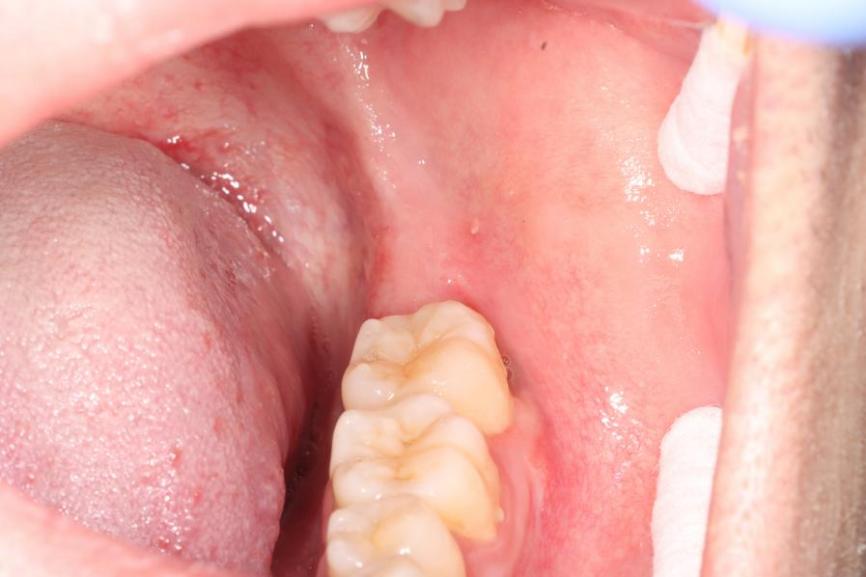


No obvious scars visible

（A follow-up examination three weeks after surgery revealed that the patient's wound had healed well, with no obvious scar tissue at the incision site. This indicates that collagen plays a positive role in promoting healing and reducing scar formation. Additionally, studies have shown that laser-assisted periodontal surgery can reduce post-operative discomfort and accelerate the healing process. Therefore, this combined therapy not only yields significant short-term results but also maintains good healing outcomes in the long term.）

**Patient feedback: The patient is a teacher. The patient reported rapid recovery after surgery and expressed satisfaction with the surgical outcome and post-operative comfort. The patient stated that the surgery did not interfere with their ability to teach students.**

**IX.Case discussion**

**Surgical characteristics：**

- 1. In traditional tooth extraction surgery, the cutting, haemostasis, and suturing of soft tissues may cause some damage to surrounding tissues, especially when dealing with complex impacted teeth or buried teeth. The application of laser technology provides more precise cutting capabilities, reducing damage to surrounding healthy tissues.
  2. Er:YAG lasers, due to their high absorption characteristics for water and hydroxyapatite, can precisely remove diseased tissues without damaging adjacent tissues. This laser has a shallow penetration depth, resulting in minimal thermal damage to surrounding healthy tissues.
  3. Nd:YAG lasers have excellent tissue penetration capabilities and are absorbed by haemoglobin and melanin, making them highly effective for soft tissue ablation and haemostasis, particularly suitable for surgical procedures within the oral cavity.

**prognosis assessment：**

- 1. Laser technology offers significant advantages in reducing post-operative swelling, pain, and infection. Due to the laser's precise cutting and coagulation effects, post-operative bleeding is minimal, and inflammatory responses are mild, thereby reducing the risk of post-operative complications.
  2. Lasers also promote the regeneration and reorganization of collagen, accelerating the wound healing process. Collagen is a crucial component of wound healing, and lasers can stimulate its production, thereby improving healing quality.
  3. Postoperative aesthetic outcomes are also an important aspect of postoperative assessment. Due to its minimally invasive nature, laser technology results in smaller and less noticeable scars postoperatively. This is a significant advantage for patients, especially those with high aesthetic expectations.

**X.Case conclusion**

The combined use of Er:YAG and Nd:YAG lasers with collagen in the extraction of impacted teeth in the middle and lower jaws has demonstrated advantages such as minimally invasive procedures, minimal bleeding, and rapid recovery, providing patients with a more comfortable and effective treatment option. In the future, laser technology is expected to find broader application in the field of oral surgery.

**Typical Case Study (2)**

**Patient Name: Huang XX Patient Age: 34 years old Patient Gender: Male**

**I.Medical History Summary**

**Chief Complaint: The patient presented with ‘recurrent swelling and pain in the left lower wisdom tooth for over six months.’**

**Present Illness: The patient presented with recurrent inflammation and swelling of the left lower wisdom tooth over the past six months, affecting chewing.**

**Past Medical History: Denies systemic diseases, no history of drug allergies.**

**Family History: No significant family medical history.**

**Personal History: No history of smoking or alcohol consumption, oral hygiene status is generally adequate.**

**II.Clinical Examination**

**Extraoral Examination:**

**Facial Contour and Colour**：No obvious asymmetry in facial contour, normal skin colour, no jaundice or pigmentation.

**Swelling and Tenderness:**No obvious swelling in the left mandibular angle region, mild tenderness on palpation, no fluctuation or crepitus.

**Facial and Maxillofacial Bones: No obvious abnormalities in bone structure, no step-off or depression, no crepitus or abnormal mobility.**

**Temporomandibular Joint: No pain during opening and closing movements, no clicking sounds, normal mouth opening range, no obvious abnormal sounds detected.**

**Facial and Neck Lymph Nodes: No obvious enlargement of submandibular or neck lymph nodes, no tenderness.**

**Intraoral examination：**

Good oral hygiene, slight redness and swelling of the gingiva throughout the mouth, no subgingival calculus or periodontal pockets detected, BOP (–), no obvious tooth mobility throughout the mouth. Tooth 38 has partially erupted, with the distal gingival flap covering it, slight redness and swelling of the gingiva, no obvious mobility. No obvious caries or periodontal lesions in the adjacent second molar.

**Other examinations:：**

**Speech: The patient's speech is clear, with no obvious impairments.**

**Salivary glands: Saliva secretion in the oral cavity is normal, with no reduction or increase, and no redness or pus discharge at the duct openings.**

**III.Imaging examination:**

**Panoramic radiograph:** The lower left third molar is horizontally impacted, with the crown in contact with the neck of the second molar. The periodontal ligament is widened, and no obvious resorption of the adjacent alveolar bone is observed. The apex is close to the inferior alveolar nerve canal.


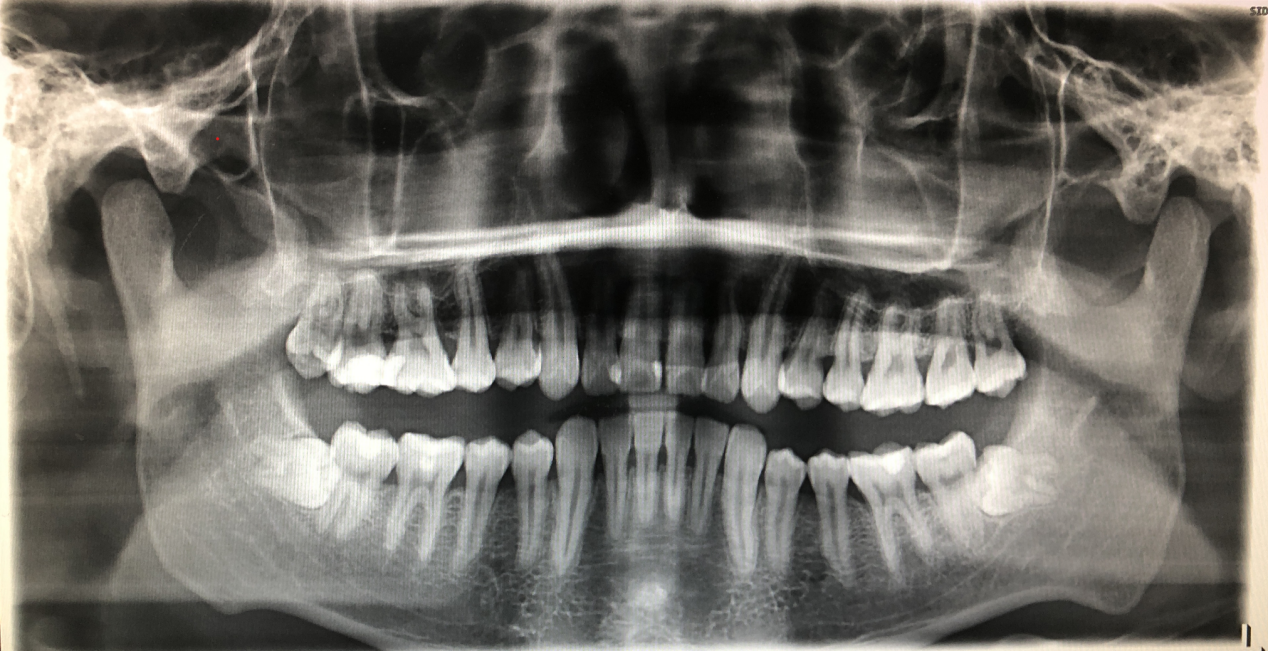


**IV.Diagnosis**

38 impacted tooth (medial mesial impaction)

**V.Treatment**

**1. Treatment plan:**

Extraction of wisdom tooth 38 to eliminate the source of infection and prevent further complications.

**2.Surgical procedure：**

- 1. First, use an Er:YAG laser (wavelength 2,940 nm) to incise the gingiva and separate the periodontal ligament. The Er:YAG laser, due to its high absorption of water and hydroxyapatite, effectively minimises thermal damage to surrounding healthy tissues while achieving precise soft tissue incisions. (Bleeding from soft tissue after Er:YAG laser excision is typically minimal, as the laser's high absorption of water and hydroxyapatite allows for precise soft tissue incisions while minimising thermal damage to surrounding healthy tissues.)
  2. Minimally invasive tooth extraction procedure: After incising and elevating the flap, an ultrasonic bone knife is used for bone removal and space creation, followed by a high-speed handpiece for tooth separation and extraction of the affected tooth.
  3. Er:YAG laser (wavelength 2,940 nm) is used again to remove granulation tissue, followed by Nd:YAG laser (wavelength 1,064 nm) for local soft tissue therapy. The Nd:YAG laser has excellent tissue penetration and is effectively absorbed by haemoglobin and melanin, making it suitable for soft tissue ablation and haemostasis.
  4. Finally, collagen protein is placed within the wound.
  5. Under the influence of the laser, the impacted tooth is successfully extracted while minimising bleeding during surgery and post-operative swelling.

**VI.Postoperative care**

- Apply ice packs immediately after surgery to reduce postoperative swelling.
- Administer antibiotics to prevent infection.
- Inform patients of postoperative precautions, including diet and oral hygiene maintenance.

**VII.Follow-up**

**Immediately after surgery:**


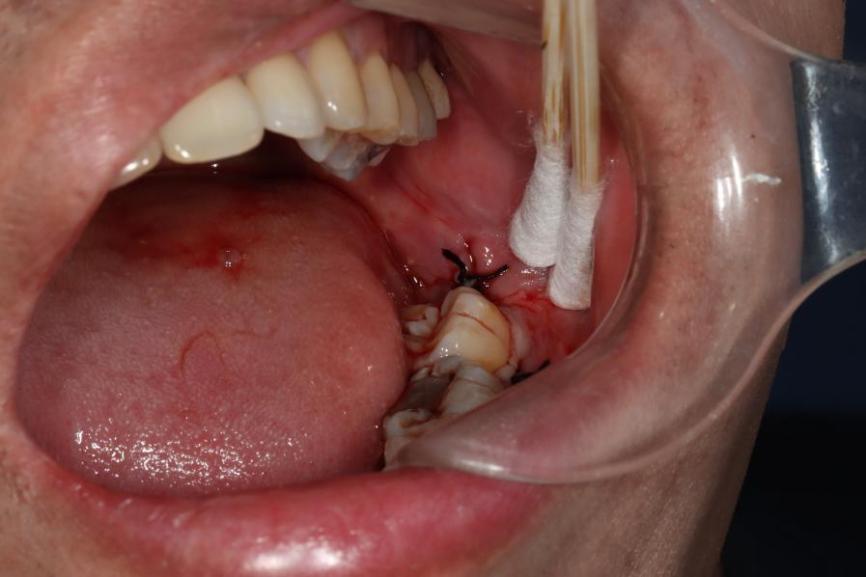


- Immediately after surgery, there was no obvious bleeding from the wound.

**3 days after surgery**：


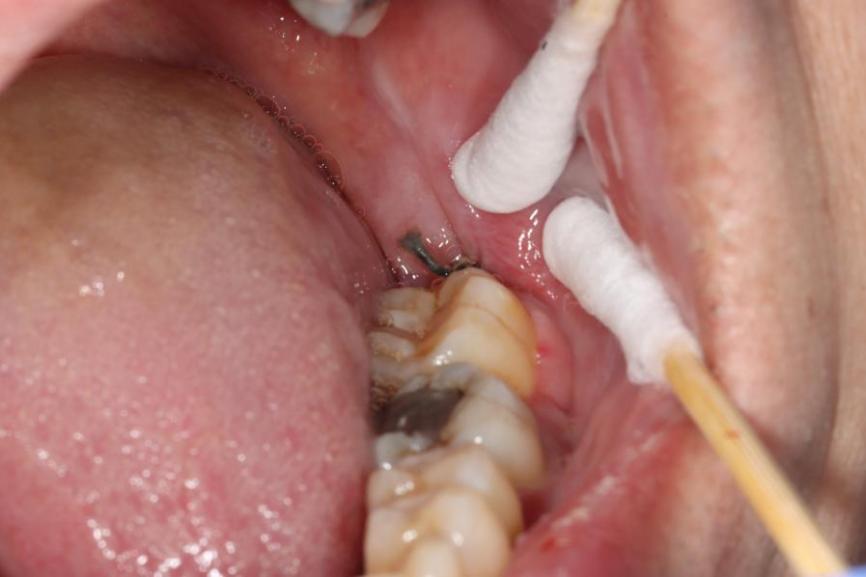


- The extraction site has healed well, with pink gums or gums that are the same colour as the surrounding area. The buccal-lingual diameter of the extraction site has shrunk significantly, and the mesial additional incision sutures have come out. There are no signs of wound dehiscence, and there is no swelling of the surrounding soft tissue. The mouth can be opened and closed freely.

**7 days after surgery**：


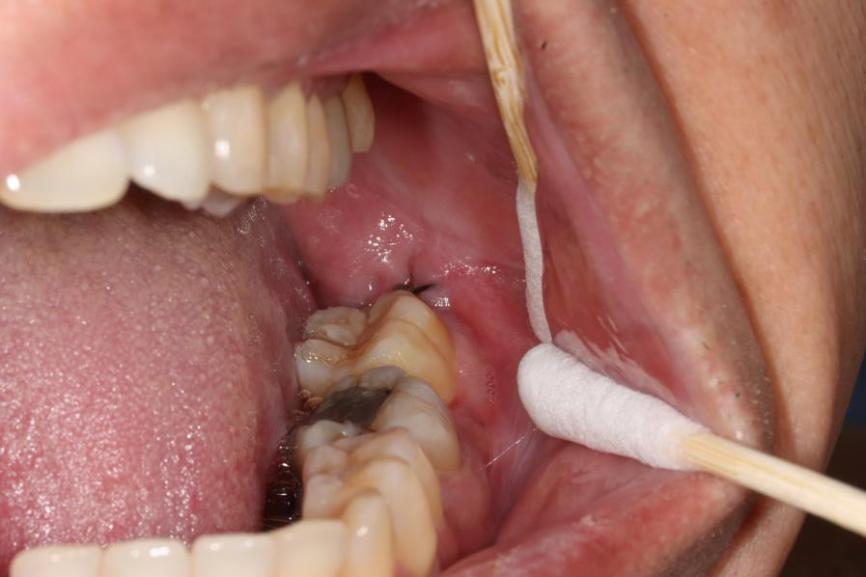


- The tooth extraction site has healed well, with pink gums or gums that are the same colour as the surrounding area.

**VIII.Case Discussion**

**• Er:YAG lasers can perform precise cutting without damaging adjacent tissues due to their high absorption characteristics for water and hydroxyapatite. This type of laser has a shallow penetration depth, resulting in minimal thermal damage to surrounding healthy tissues.**

**• Nd:YAG lasers have excellent tissue penetration capabilities and are absorbed by haemoglobin and melanin, making them highly effective for soft tissue ablation and haemostasis.**

**• Laser technology offers significant advantages in reducing post-operative swelling, pain, and infection. Due to the laser's precise cutting and coagulation effects, post-operative bleeding is minimal, and inflammatory responses are mild, thereby reducing the risk of post-operative complications.**

**• Lasers also promote the regeneration and reorganization of collagen, accelerating the wound healing process. Collagen is a crucial component of wound healing, and lasers can stimulate its production, thereby improving healing quality.**

**• Collagen scaffolds form a stable network of cross-linked structures, facilitating cell migration and proliferation, and promoting tissue growth and repair**

**IX. Conclusion**

**The combined use of Er:YAG laser and Nd:YAG laser with collagen protein in the extraction of impacted teeth in the middle and lower positions has demonstrated advantages such as minimally invasive surgery, minimal bleeding, reduced postoperative swelling and mouth opening, and good wound healing, providing patients with a more comfortable and effective treatment option.**

**Appendix II Clinical trial number**


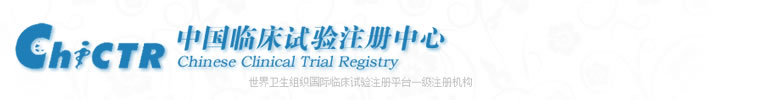


**Clinical trial number:** PID:248812

**Appendix III Competing interests**

Declaration of Interest – Principal Investigator

| Research Project | **Er:YAG and Nd:YAG lasers with medical collagen improve third-molar extraction wound healing: a randomized controlled trial** |
| --- | --- |
| Project Source | Hospital-Level Scientific Research Project, Hospital of Stomatology, Zhongshan City. |

I understand that I, my spouse, dependent children, or business partners must voluntarily disclose any financial interests—of any amount—related to the research project or its sponsor.

In accordance with the institutional policy on research conflicts of interest, I hereby declare the following financial interests concerning the above project:

| Patent licensing or transfer of research results to the project sponso | 🞎YES，🗹NO |
| --- | --- |
| Investment relationship with the project sponsor | 🞎YES，🗹NO |
| Purchase, sale, or lease of any property or real estate with the project sponsor | 🞎YES，🗹NO |
| \| Financial interest in a competing product similar to the study product \|  \| \| --- \| --- \| | 🞎YES，🗹NO |
| Employment or service relationship with the project sponsor | 🞎YES，🗹NO |
| Receipt of consultant/advisory fees from the project sponsor (specify amount if yes) | 🞎YES，🗹NO |

| Commitment | The above declaration of conflicts of interest is true and accurate. |
| --- | --- |
| Signature |  |
| Date |  |

### ****Appendix IV** Consent to participate**

### Consent to Participate（Chinese-English Bilingual）

English version
All procedures performed in this study involving human participants were in accordance with the ethical standards of the Hospital of Stomatology,Zhongshan City Clinical Research & Laboratory Animal Ethics Committee (approval No.【2024】Research Project No. 7, dated [March 18, 2024]) and with the 1964 Helsinki Declaration and its later amendments or comparable ethical standards. Written informed consent was obtained from every individual participant (or from the parent/legal guardian for participants under 18 years of age) after a full explanation of the study aims, procedures, potential risks and benefits, as well as the right to withdraw at any time without prejudice. A copy of the signed consent form was provided to each participant/guardian and another copy is retained in the hospital’s confidential medical record.

中文版本
本研究所有涉及人的操作均已获得中山市口腔医院临床科研与实验动物伦理委员会批准（批准号：【2024】科研第（7）号，批准日期：[2024年3月 18日]），并严格遵循 1964 年《赫尔辛基宣言》及其后续修订案或同等伦理规范。研究开始前，研究者已向每位受试者（或对 18 岁以下受试者的家长/法定监护人）充分说明了研究目的、流程、潜在风险与收益以及随时可退出且不受歧视的权利，并由其本人（或其监护人）签署书面知情同意书。签署的同意书一份交予受试者/监护人保存，另一份存档于医院保密病历中。

**知情同意书（中文版本）**

尊敬的患者：

您被邀请参加铒激光、钕激光联合胶原蛋白辅助阻生第三磨牙的治疗，医生为张同韩、袁展棠。本须知提供给您一些信息以帮助您决定是否接受治疗。请您仔细阅读，如有任何疑问请向医生提出。

您参加本项治疗是自愿的，我们将和您或您的家人进行详细沟通，向您介绍该项治疗的有关情况，也请您提供与疾病有关的情况，包括发病过程、家族史、以前就诊情况及曾经做过哪些检查和治疗。

风险与不适：对于您来说，与我们进行沟通、交谈可能会有些心理不适。

受益：通过对您的信息资料进行收集，将对疾病诊治、生物医学科学研究等提供重要的证据，产生一定的社会价值。

个人信息及隐私保护：您的个人资料均属保密，未经您授权我们不会将您个人信息及隐私向第三方透露。

费用：按照三级医院收费标准收取激光治疗费、医用胶原蛋白海绵材料费和药费，其余免费。

知情同意声明：

我已被告知此项治疗的目的、背景、过程、风险及获益等情况。

我有足够的时间和机会进行提问，问题的答复我很满意。

我已经阅读了本知情同意书。

患者姓名：________________________联系电话：________________________

患者（或代理人）签名：_________________________（ 关系）

日期：______ _年________月________日

我已准确地将这份文件告知者，他/她准确地阅读了这份知情同意书，并有机会提出问题。

研究者姓名：________________________联系电话：________________________

研究者签名：_________________________

日期：______ _年________月_____

**Informed Consent Document（English version）**

Dear Patient,

You are being invited to participate in a therapeutic procedure involving the use of Er:YAG and Nd:YAG lasers, in conjunction with collagen, for the treatment of impacted third molars. The attending physicians are Dr. Zhang Tong-Han and Dr. Yuan Zhan-Tang. This document is intended to provide you with essential information to assist you in making an informed decision regarding your participation. Please review it thoroughly and consult with the physician if you have any inquiries.

Your involvement in this treatment is entirely voluntary. We will engage in comprehensive communication with you or your family, detailing the treatment's specifics, and we request that you share pertinent medical history, including the disease's progression, familial medical background, prior medical consultations, and any diagnostic tests or treatments you have previously undergone.

Risks and Potential Discomfort: Engaging in dialogue with us for the purpose of this communication may induce some psychological distress.

Benefits: The collection of your medical information will contribute significantly to the diagnosis, treatment, and biomedical research of diseases, thereby generating societal value.

Confidentiality of Personal Information and Privacy Protection: The confidentiality of your personal data is assured. We will not disclose your personal information or privacy to any third party without your explicit consent.

Costs: Fees for laser therapy, medical collagen sponge materials, and pharmaceuticals will be levied in accordance with the tariff of a tertiary care hospital, with all other costs being waived.

Statement of Informed Consent:

I have been apprised of the objectives, context, procedures, risks, and benefits associated with this treatment.

I have been afforded ample time and opportunity to pose questions, and I am satisfied with the responses received.

I have read and understood this Informed Consent Document.

Patient's Name: ___________________________

Contact Number: _____________________________

Signature of Patient (or Legal Representative): _______________________ (Relationship)

Date: _______ Year _______ Month _______ Day

I have accurately communicated the contents of this document to the aforementioned individual, who has read and comprehended this Informed Consent Document and has had the opportunity to raise questions.

Investigator's Name: ___________________________

Contact Number: _____________________________

Signature of Investigator: _________________________

Date: _______ Year _______ Month _______ Day

**Appendix V Funding Declaration**

**Funding Declaration**

This work was supported by Hospital of Stomatology, Zhongshan City.The funding body had no role in the design of the study, collection, analysis, interpretation of data, or writing of the manuscript.

**Appendix VI Ethics approval**

**Ethics approval**

This study was approved by the **Hospital of Stomatology,Zhongshan City Clinical Research & Laboratory Animal Ethics Committee** (approval No.【2024】Research Project No. 7, dated [March 18, 2024]) and was conducted in accordance with the 1964 Declaration of Helsinki and its later amendments.
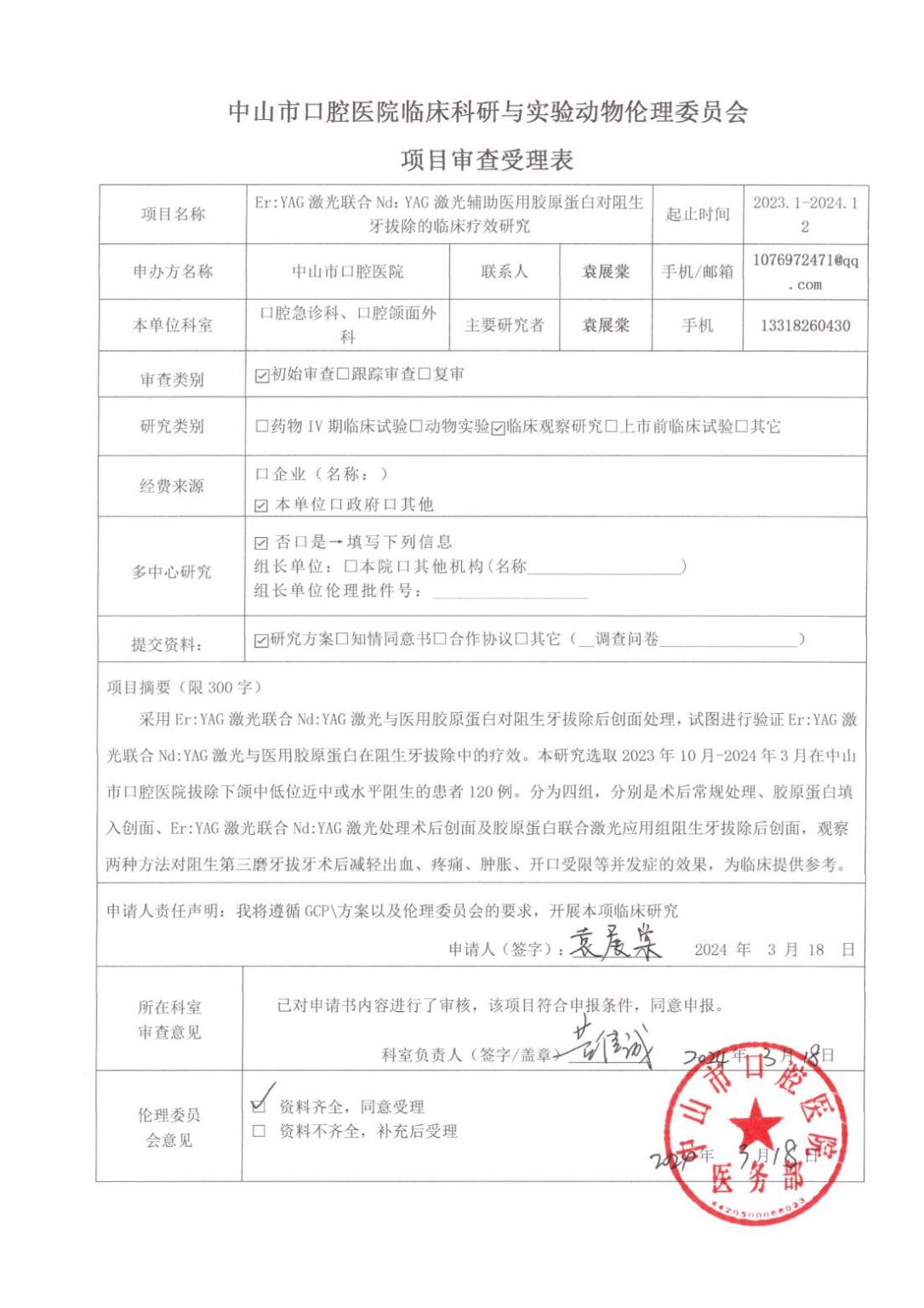

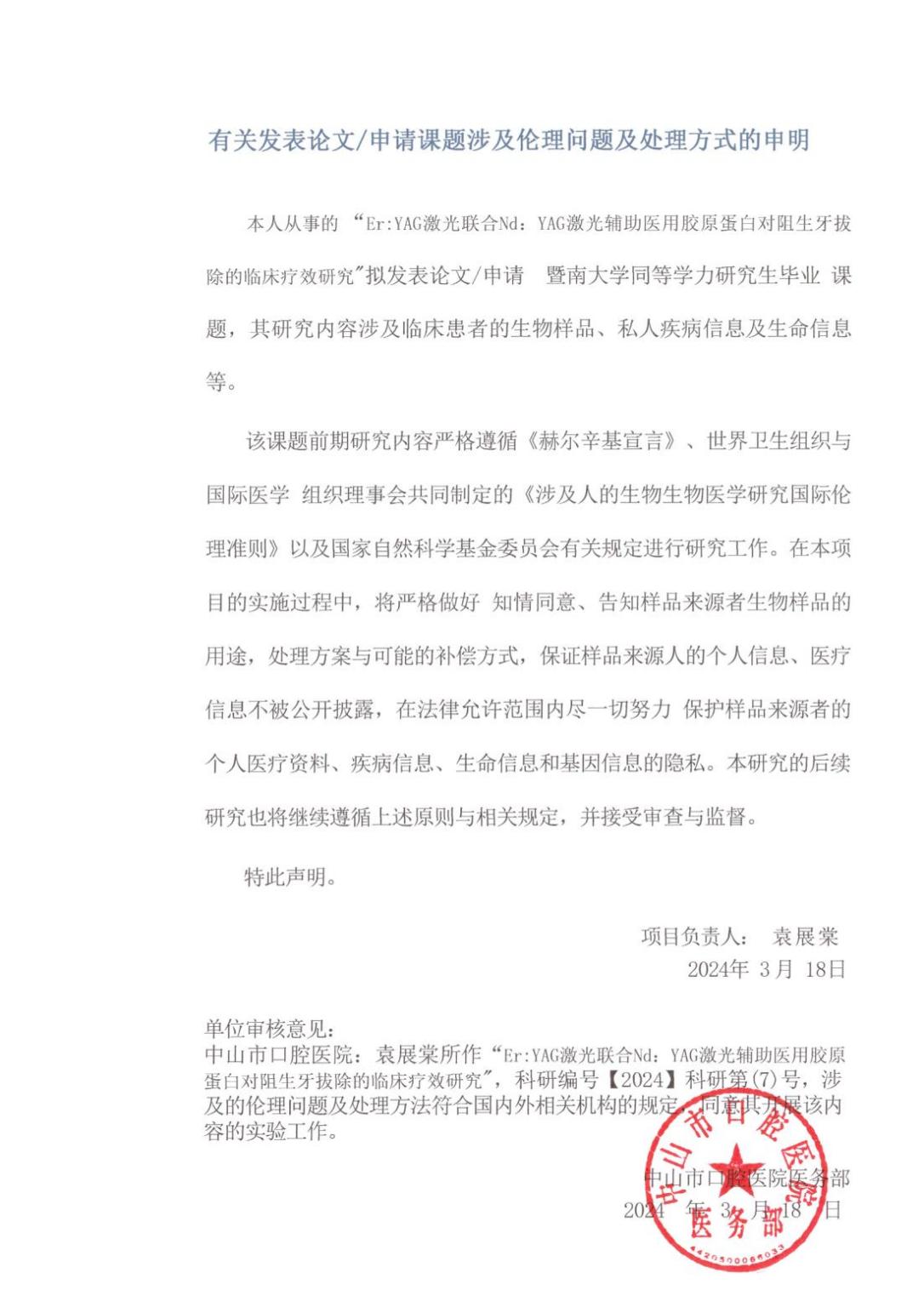

Supplement: Supplementary file 1 — Supplementary Material 1 (DOCX 1.17 MB) [file 10103_2025_4763_MOESM1_ESM.docx]
