# Supplementary figures and images for: Er:YAG and Nd:YAG-based low-level laser therapy (LLLT) with medical collagen improve third-molar extraction wound healing: a randomized controlled trial
Source: Lasers Med Sci. 2025 Dec 8;40(1):512. doi: 10.1007/s10103-025-04763-7 (PMC12682916; doi:10.1007/s10103-025-04763-7)

**Appendix V Availability of data and materials**


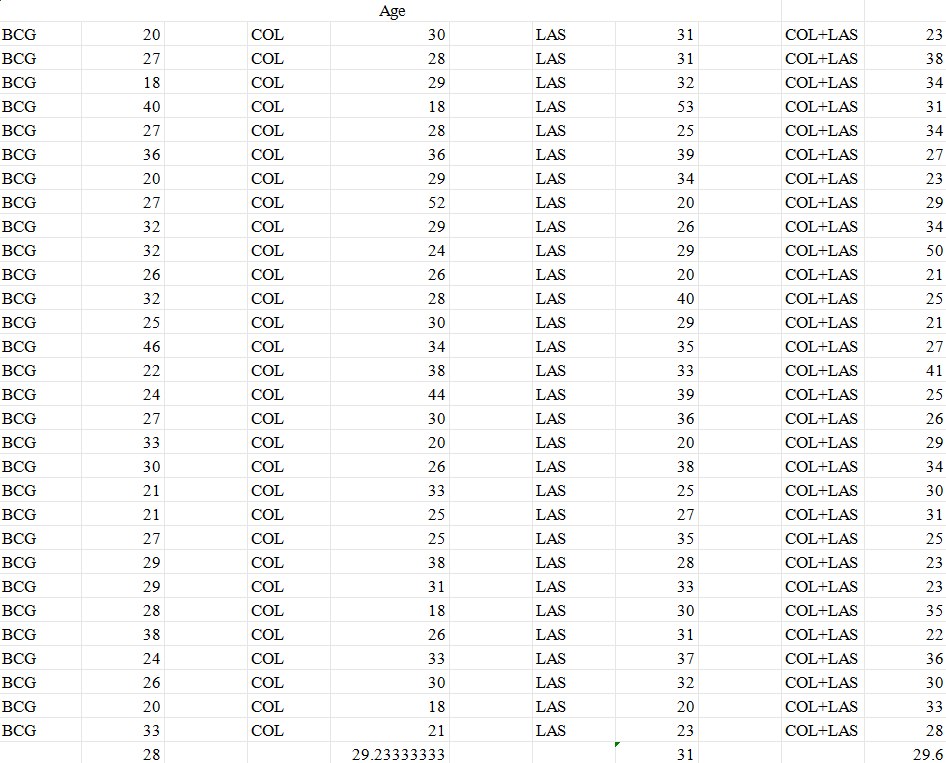

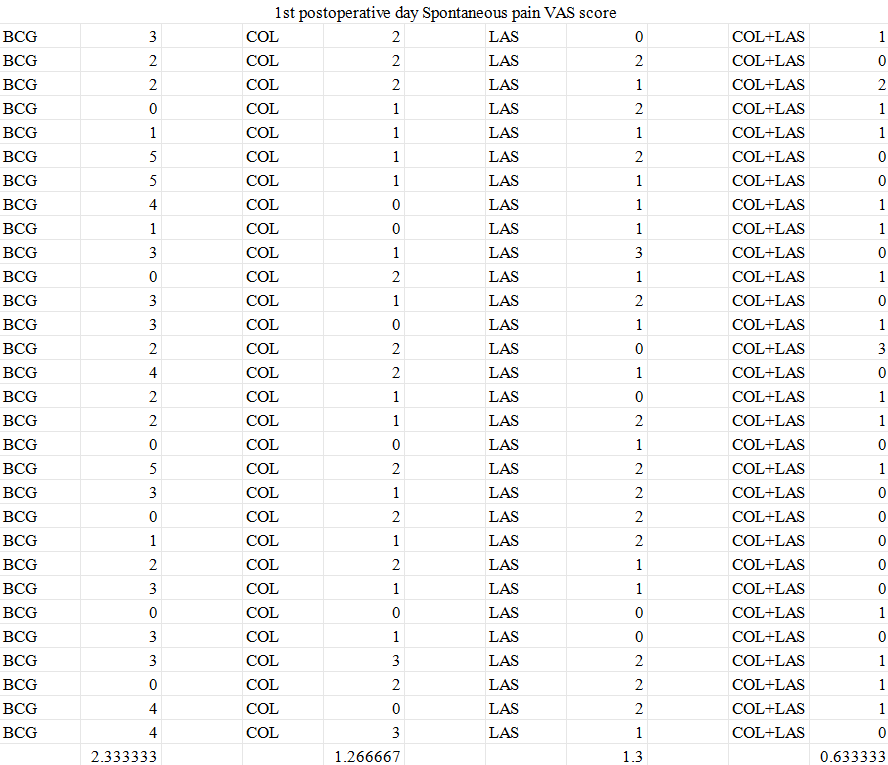

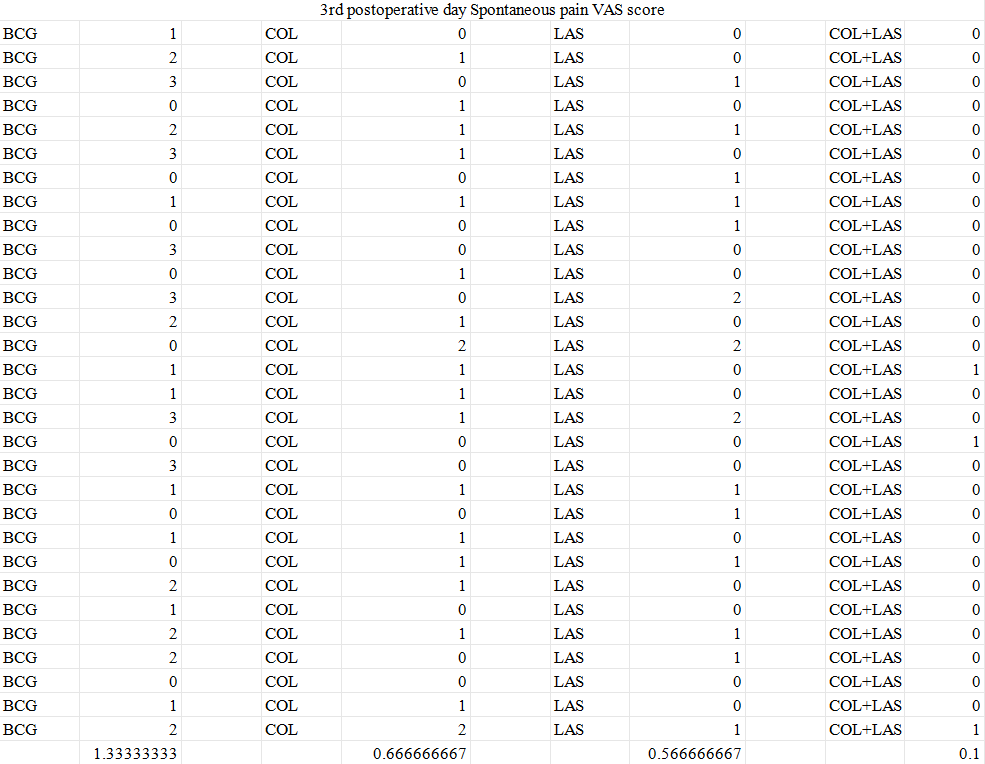

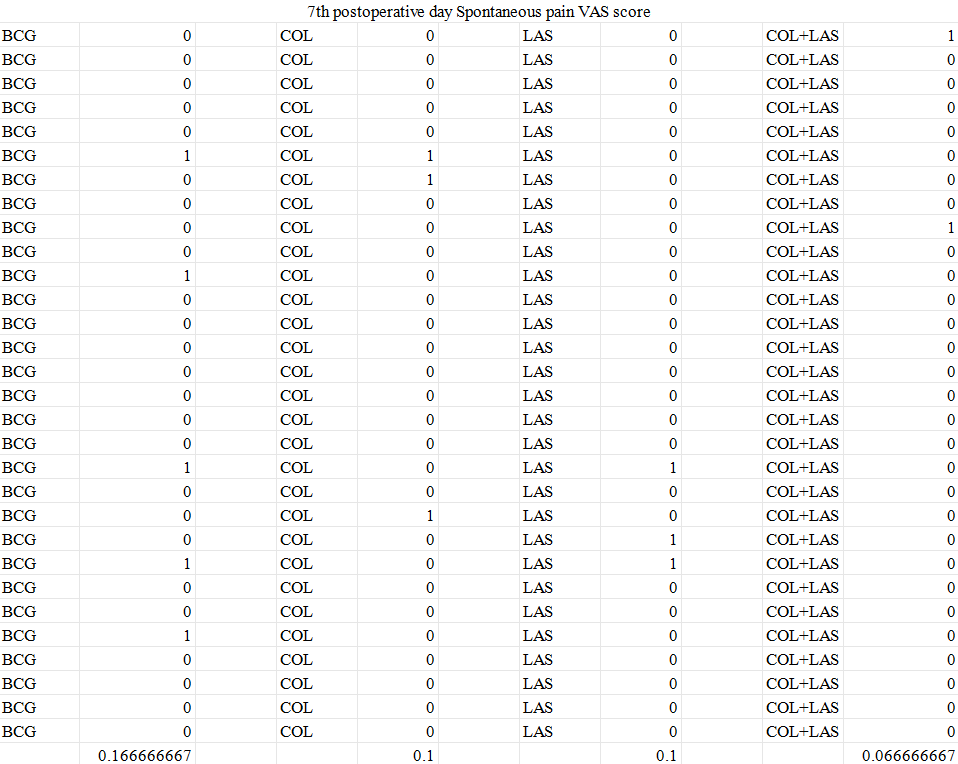

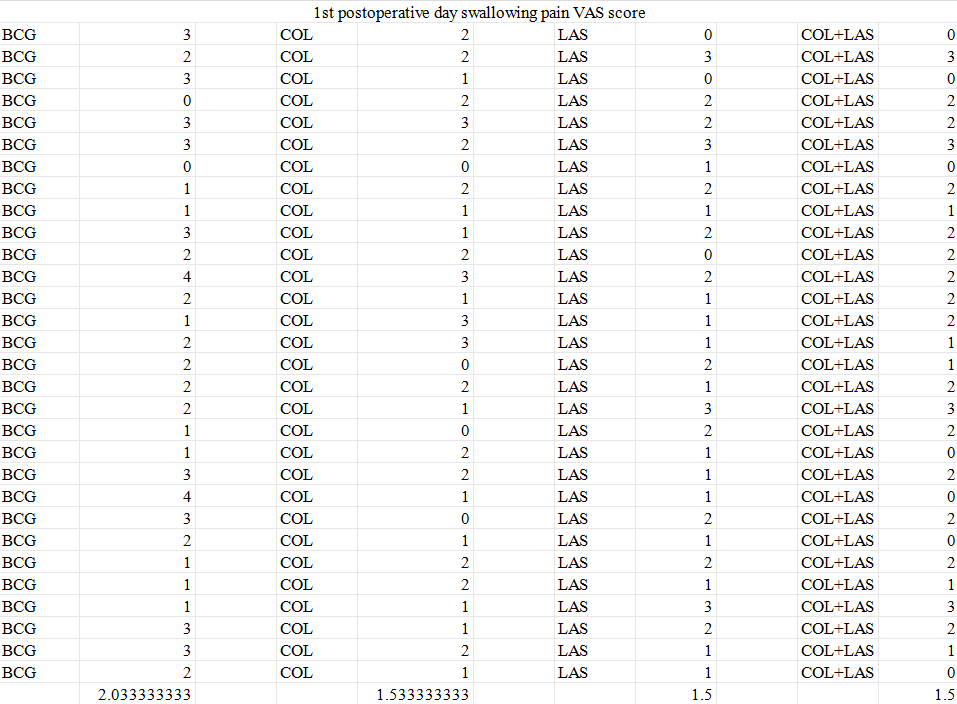

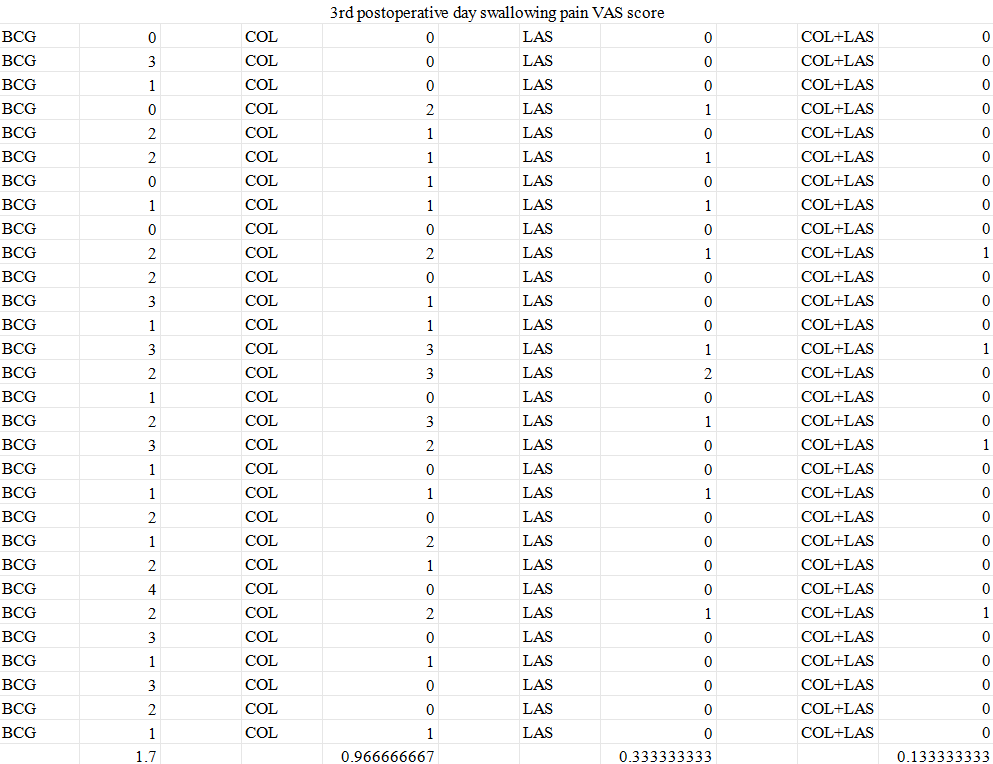

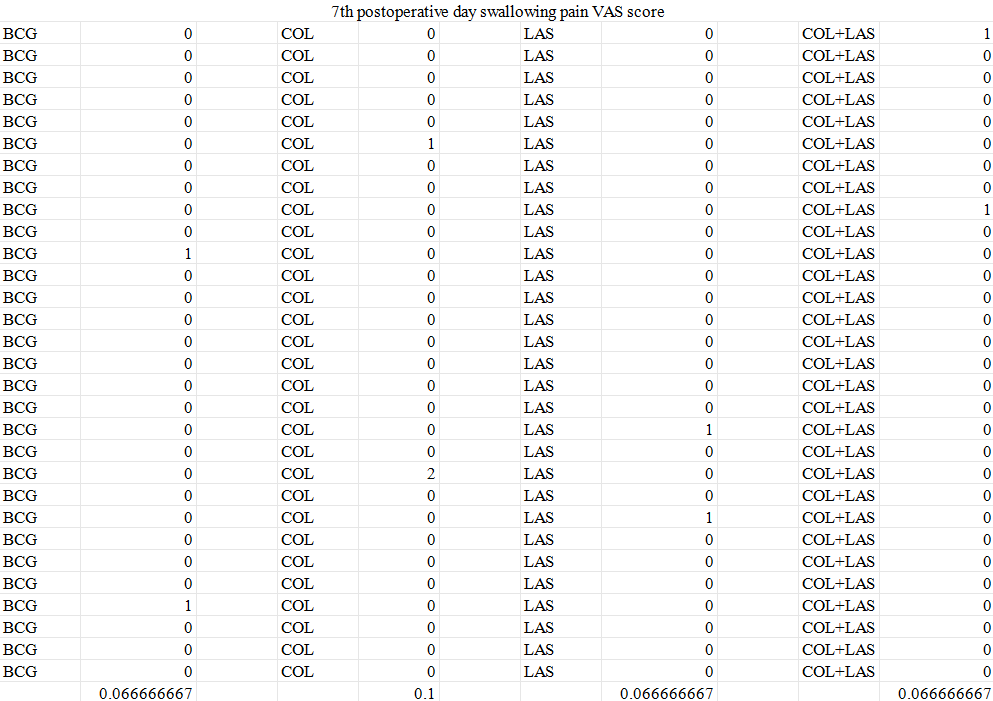

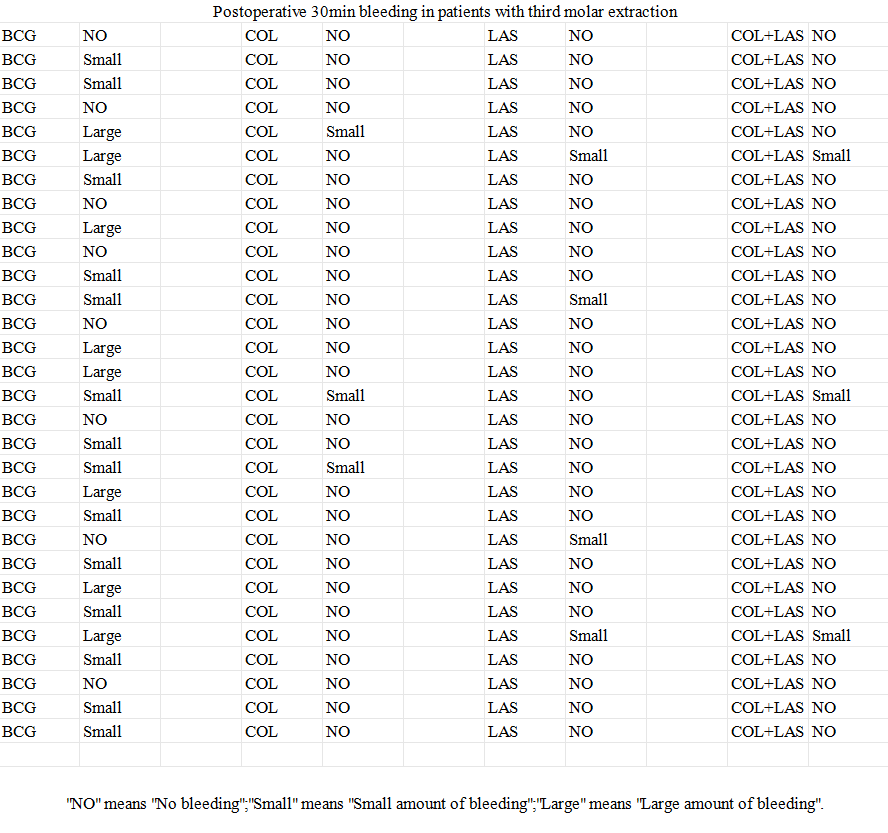

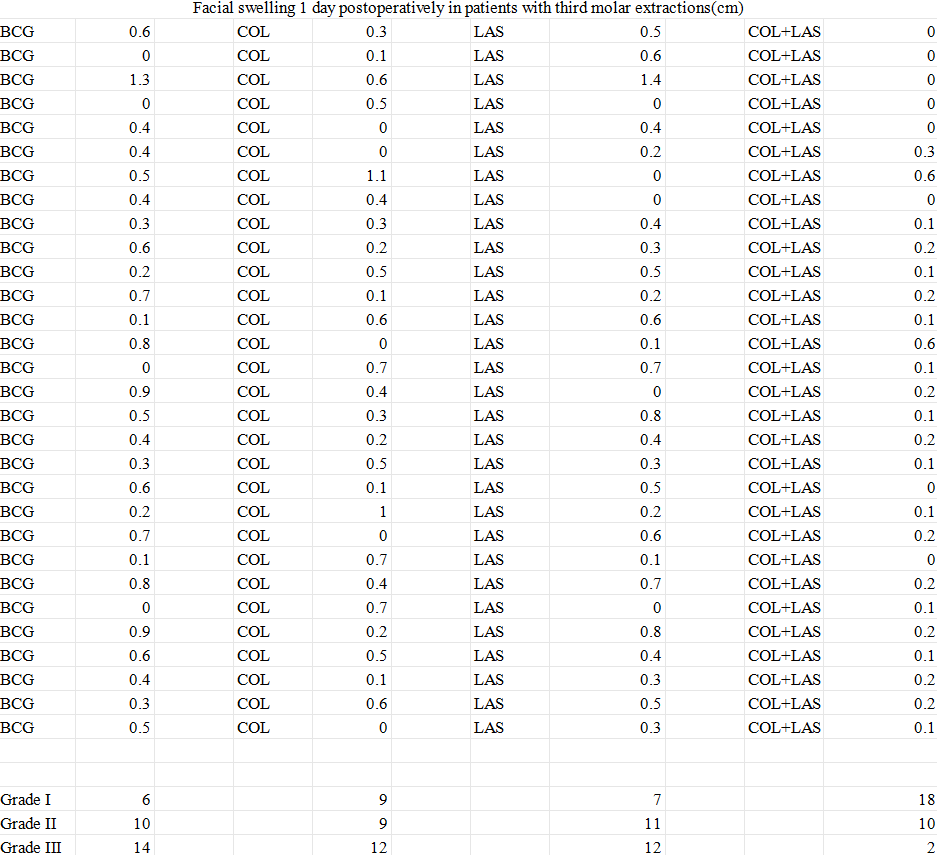

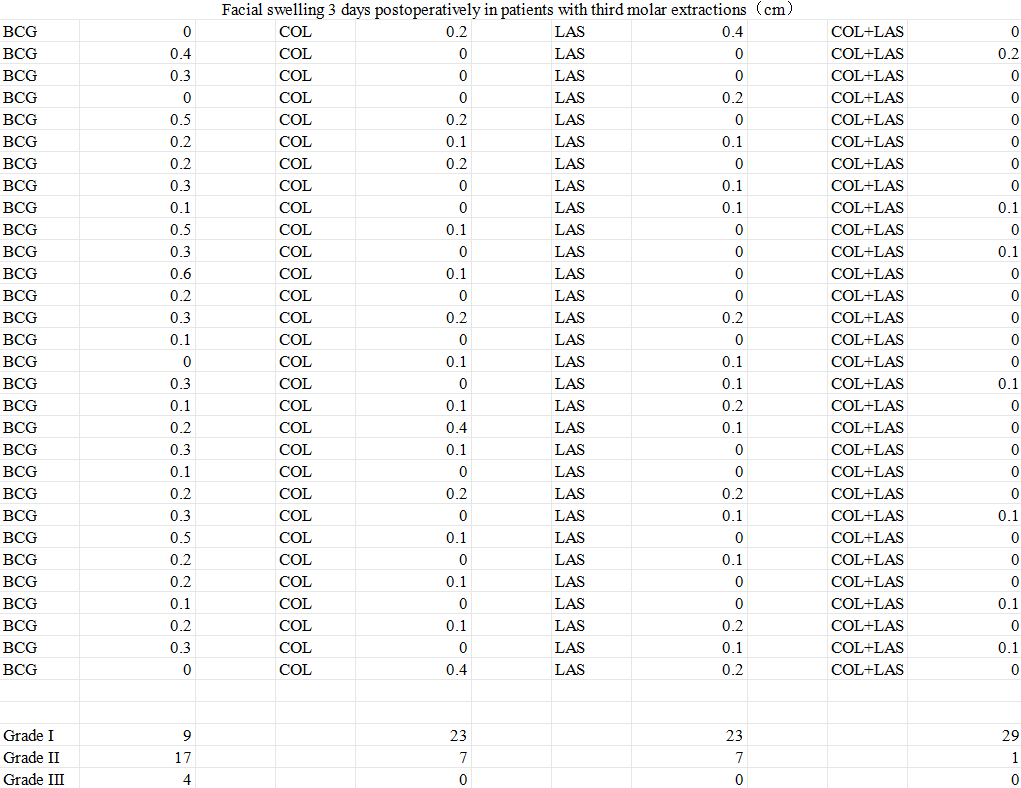


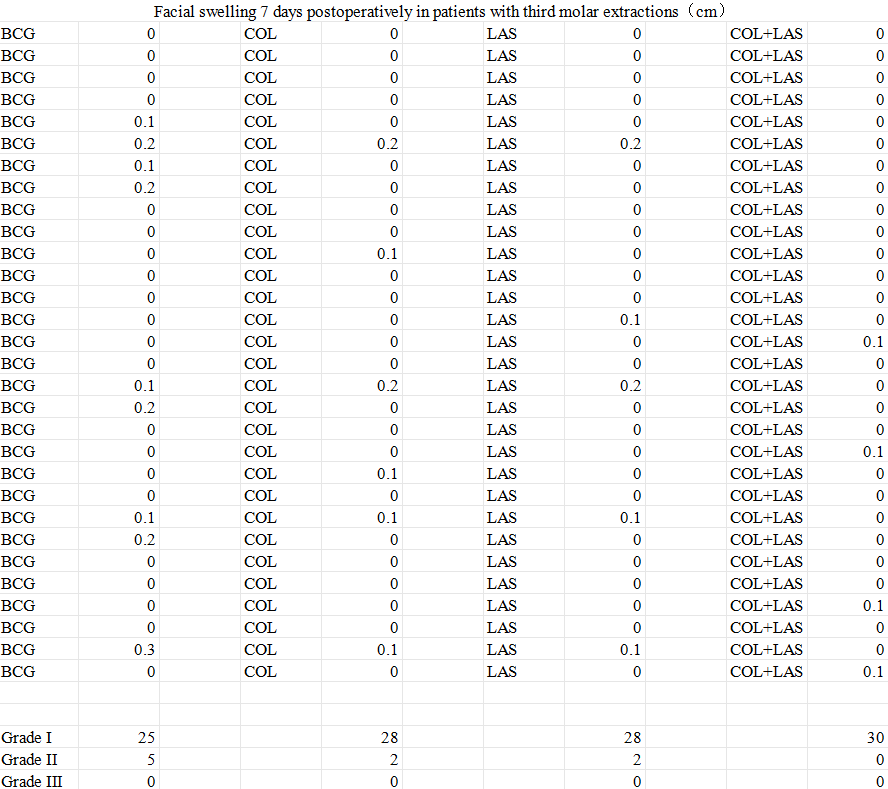

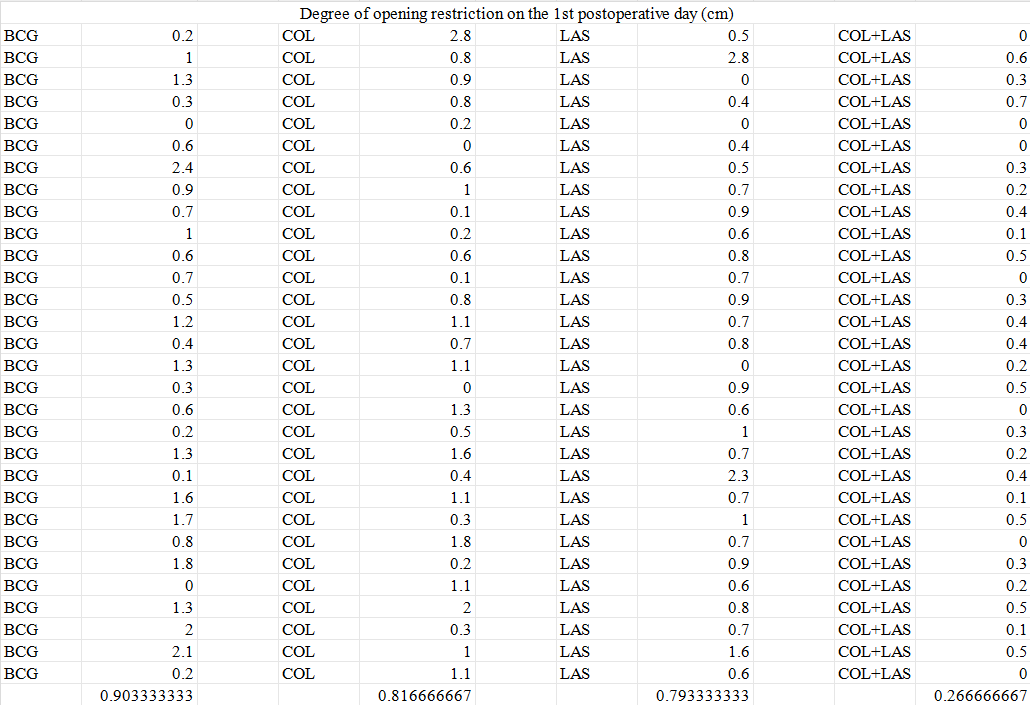

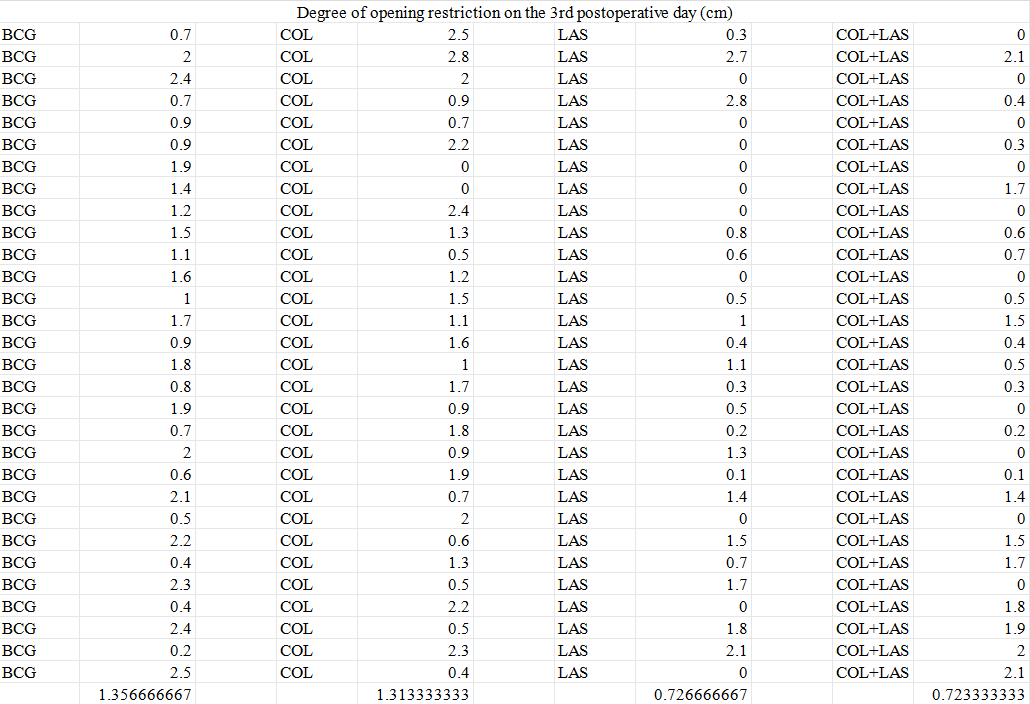

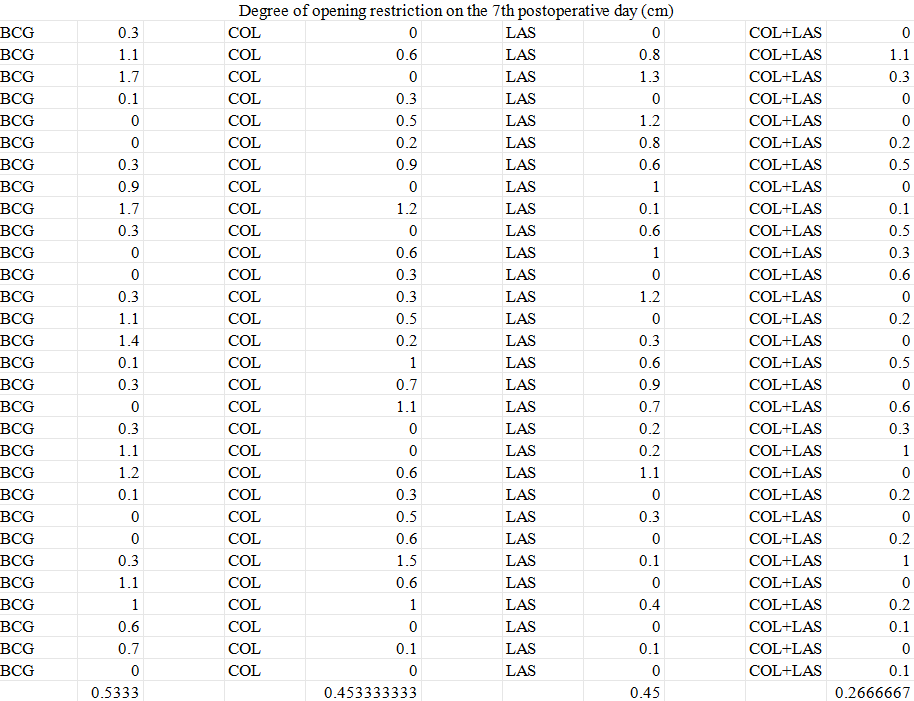


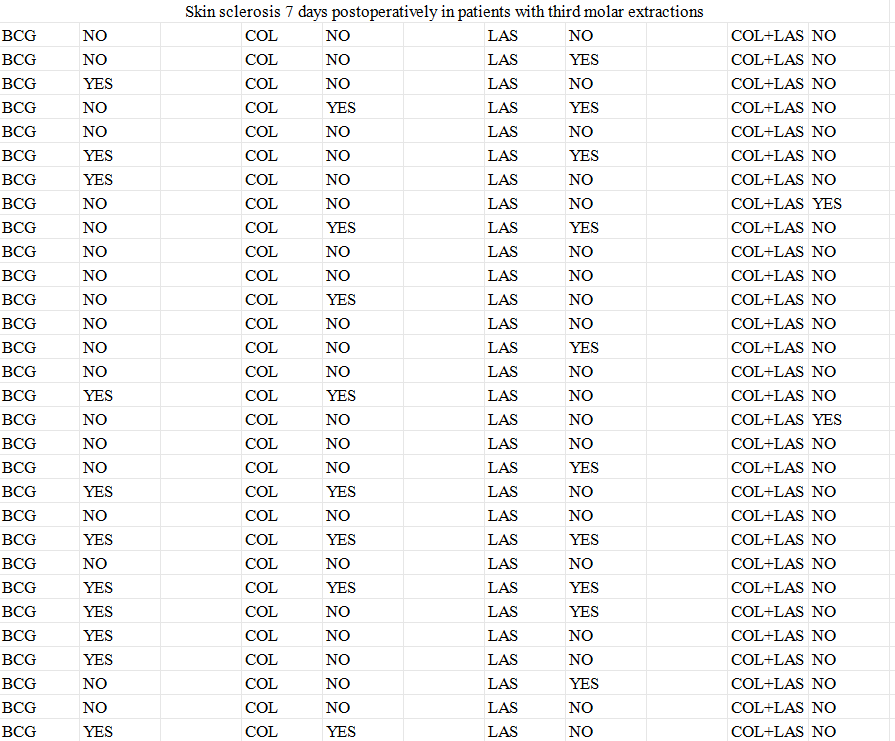

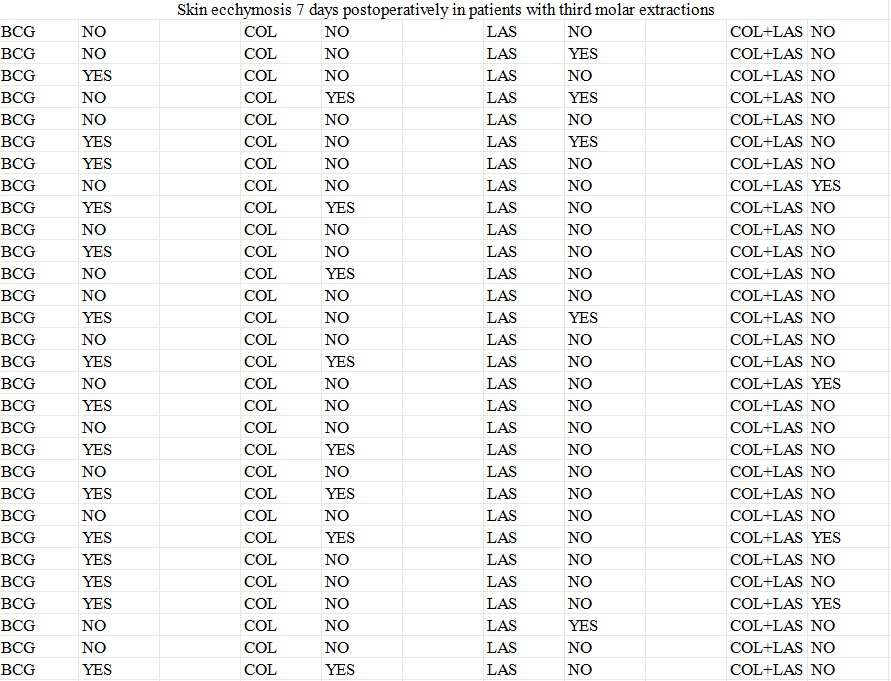

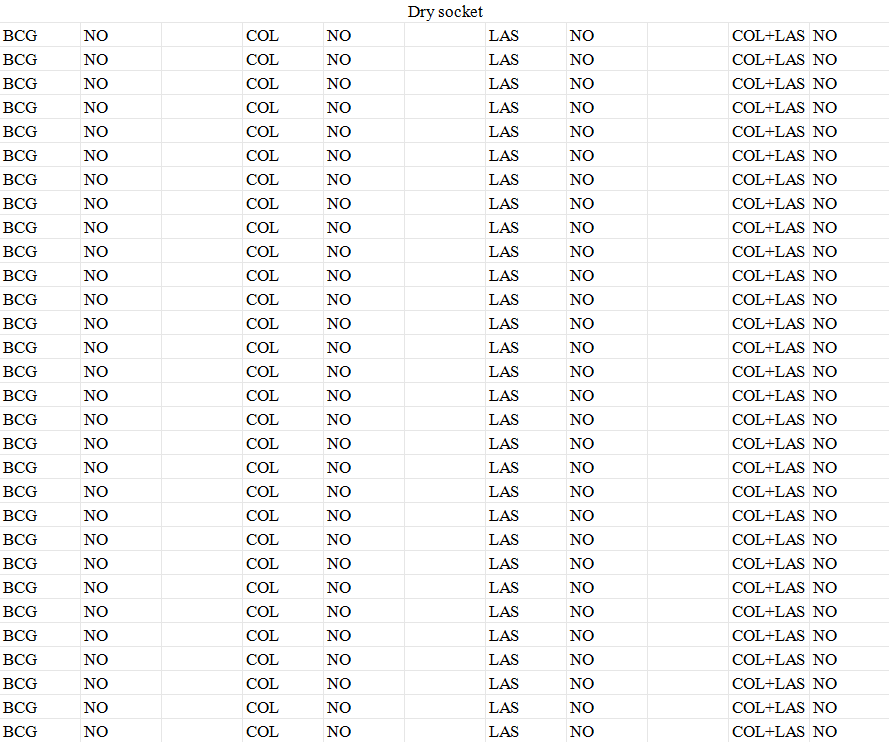

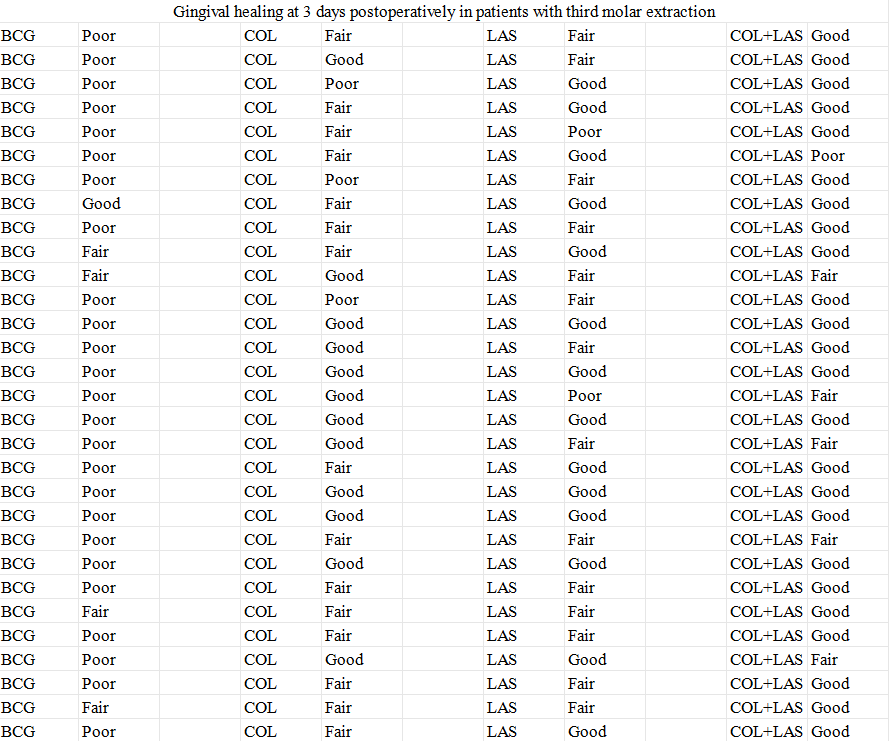

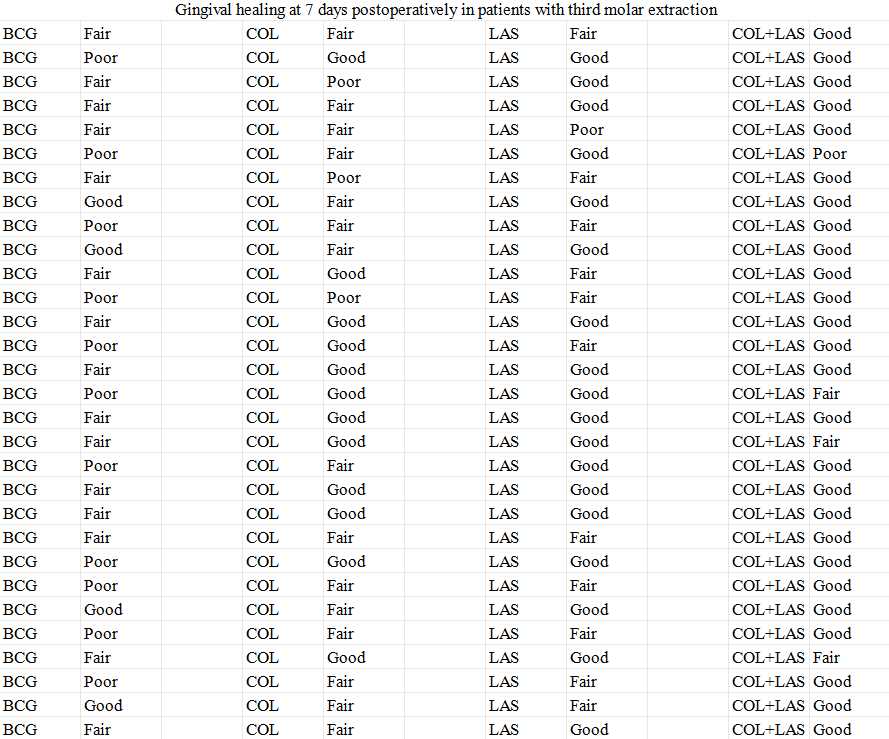

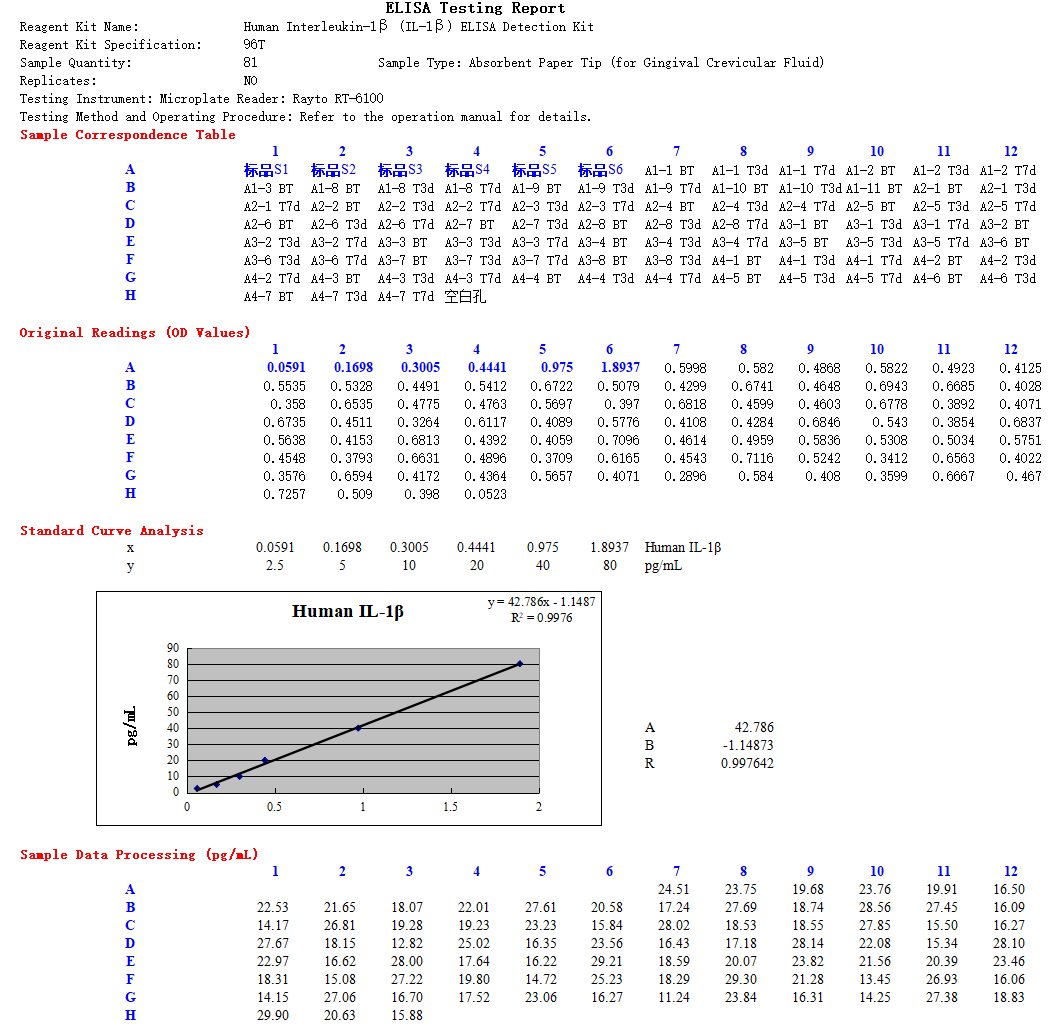

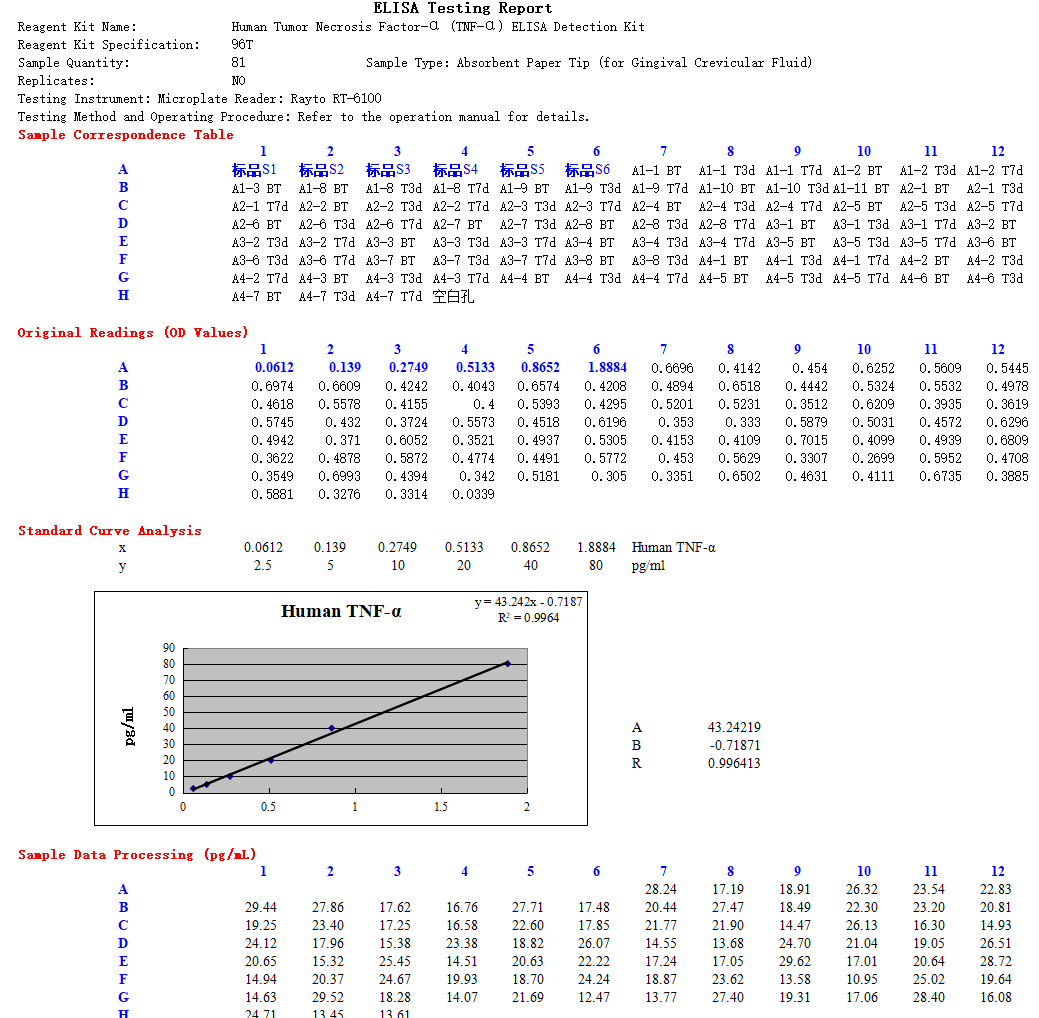

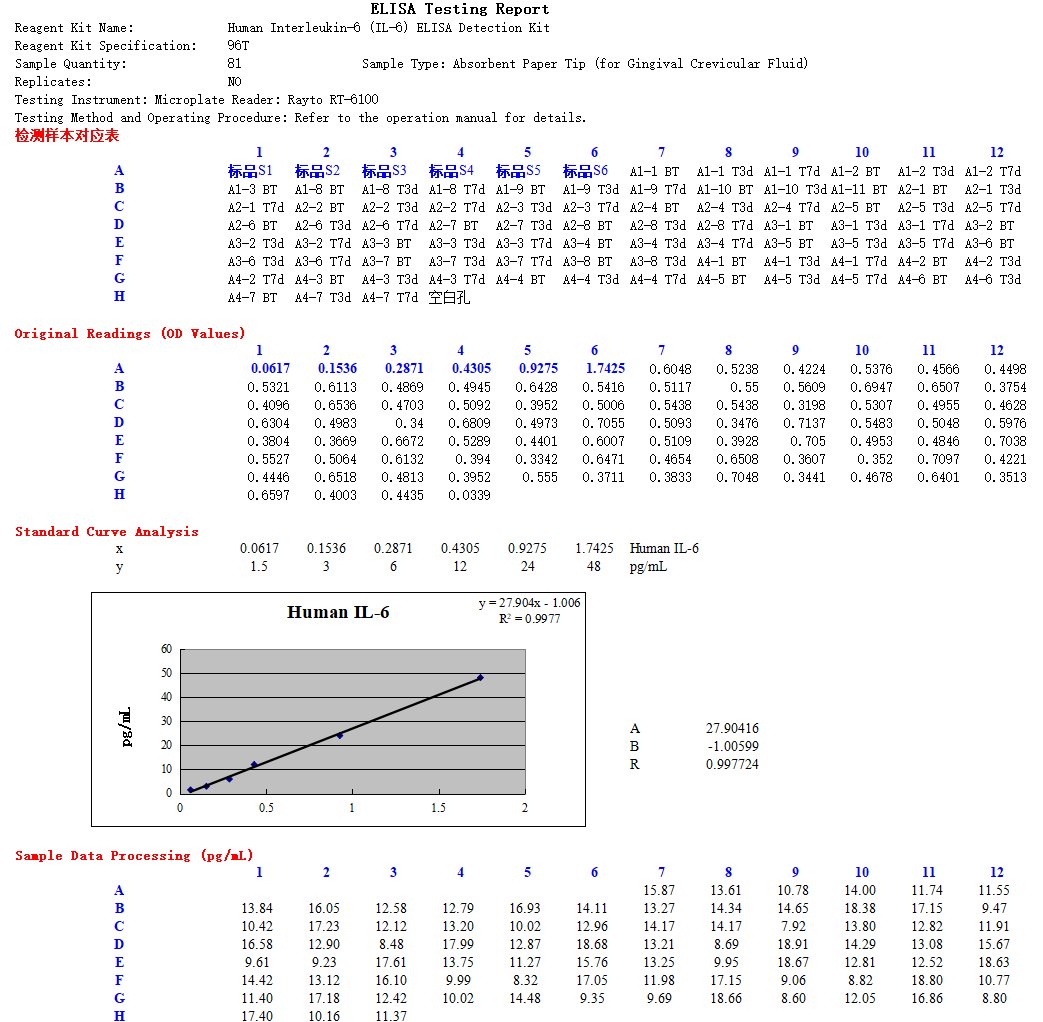

Supplement: Supplementary file 2 — Supplementary Material 2 (DOCX 1.17 MB) [file 10103_2025_4763_MOESM2_ESM.docx]
